# Integrated network pharmacology and bioinformatics analysis reveals multi-target mechanisms of HeJie Shengfa Decoction against alopecia areata

**DOI:** 10.7717/peerj.21006

**Published:** 2026-07-14

**Authors:** Lingling Zhao, Haixia Mi, Zhaokun Diao, Aikeda Tiemuer, Limin Mou, Jingxuan Yang, Lingmeng Zuo, Chenghui Zhang

**Affiliations:** 1 The Fourth Clinical Medical College of Xinjiang Medical University, Urumqi, Xinjiang, China; 2 Wenzhou People’s Hospital (The Wenzhou Third Clinical Institute Affiliated To Wenzhou Medical University), Wenzhou, Zhejiang, China; 3 Xinjiang Uygur Autonomous Region Hospital of Traditional Chinese Medicine, Urumqi, Xinjiang, China

**Keywords:** Alopecia areata, Traditional Chinese medicine, Hejie Shengfa decoction, Network pharmacology, Bioinformatics

## Abstract

**Background:**

Alopecia areata (AA) is a common non-scarring autoimmune disease with a complex pathogenesis, high recurrence rates, and difficulty in achieving a cure. Recent years have seen an increasing focus on both clinical and basic research regarding AA. Hejie Shengfa Decoction (HSD), a traditional Chinese medicine formula, has shown certain efficacy in the clinical treatment of AA, though its underlying mechanisms remain unclear.

**Methods:**

In this study, we retrieved the active ingredients of HSD and their associated targets from public databases. We then identified AA-related targets through bioinformatics analysis of publicly available AA datasets. A protein-protein interaction (PPI) network was constructed to generate the HSD-AA action network by integrating drug-specific targets and disease-related targets. Functional enrichment analysis was subsequently performed. To further investigate key genes, three machine learning algorithms—least absolute shrinkage and selection operator (LASSO) regression, support vector machine-recursive feature elimination (SVM-RFE), and Random Forest—were applied Additionally, immune cell infiltration analysis was carried out to examine the roles of these key genes in the local immune microenvironment. Molecular docking and molecular dynamics simulations were employed to assess the binding stability of the active ingredients with the core targets.

**Results:**

The results revealed that HSD contains 96 active ingredients and 985 related targets. From the AA dataset, 955 differentially expressed genes and 492 co-expressed modular genes were identified, resulting in 23 intersecting genes after integration. Machine learning algorithms identified four key target genes among these 23. Immune infiltration analysis suggested that HSD could influence the immune microenvironment of AA by modulating the expression of these key targets. Molecular docking and molecular dynamics simulations confirmed strong and stable binding interactions between HSD’s main active ingredients—especially quercetin—and the core targets.

**Conclusion:**

This study elucidates the potential of HSD as a treatment for AA and provides insights into its mechanisms of action, offering a novel approach for treating AA with multi-targeted traditional Chinese medicine.

## Introduction

Alopecia areata (AA) is a prevalent autoimmune disorder characterized by sudden, patchy hair loss, which, in severe cases, can progress to complete scalp alopecia or even total body hair loss. The global prevalence of AA is estimated at approximately 2%, with an increasing incidence observed particularly in younger populations (Seneschal, Boniface & Jacquemin, 2022). Beyond its physical manifestations, AA significantly impacts patients’ mental health, social functioning, and overall quality of life, frequently contributing to depression and anxiety (McMichael & Roberson, 2023). Although the exact pathogenesis of AA remains incompletely understood, it is widely believed to involve a multifactorial interplay of genetic predisposition, immune dysregulation, oxidative stress, microbiome imbalances, allergic reactions, and epigenetic modifications (Zhou et al., 2021; Šutić Udović et al., 2024). AA is classically recognized as a T-cell-mediated, follicle-specific autoimmune disorder, precipitated by these complex interactions (Simakou et al., 2019). Moreover, sleep disturbances, commonly reported among AA patients, exacerbate the condition, creating a vicious cycle of hair loss and emotional distress (Xie, Sun & Song, 2022; Park et al., 2023). Currently, no definitive cure exists for AA. Clinical interventions, including topical steroids, intralesional steroids, immunotherapy, Janus kinase (JAK) inhibitors, and minoxidil, show varying degrees of efficacy; however, they are often associated with adverse effects, drug resistance, and relapse upon cessation of treatment (Strazzulla et al., 2018; Alhanshali et al., 2023; Yan et al., 2022). As a result, there is a pressing need for the development of more effective and safer therapeutic strategies. Early intervention is crucial to control disease progression, improve quality of life, and alleviate the psychological burden on patients.

In recent years, traditional Chinese medicine (TCM) has attracted increasing attention in the treatment of alopecia areata (AA) because of its potential to enhance hair follicle function, promote hair regeneration, and reduce hair loss (He et al., 2021; Lv et al., 2022; Zhang et al., 2022; Li et al., 2022a; Li et al., 2025; Zhang et al., 2025). The therapeutic potential of TCM is mainly attributed to its multi-component, multi-target synergistic actions, together with a generally low toxicity profile (Zhang et al., 2022). Hejie Shengfa Decoction (HSD) was developed by Professor Chenghui Zhang, a Qihuang Scholar from the Department of Dermatology at the Affiliated Hospital of Traditional Chinese Medicine, Xinjiang Medical University, based on many years of clinical experience, and has been applied in clinical practice for patients with alopecia areata (AA). HSD is theoretically derived from the classical TCM prescription Chaihu Jia Longgu Muli Decoction recorded in Shang Han Lun (Treatise on Febrile Diseases), and was modified according to the pathogenesis characteristics of AA. This classical formula is traditionally associated with the syndrome pattern of “chest oppression with vexation and fright” (He et al., 2021). Patients with AA frequently present with anxiety, depression, and sleep disturbances, which correspond to the TCM concepts of “vexation” and “fright,” suggesting a theoretical consistency between the syndrome pattern and the clinical manifestations of AA. In recent years, Chaihu Jia Longgu Muli Decoction has been increasingly applied in AA and other dermatological diseases in clinical settings (Lv et al., 2022). HSD consists of ten herbal ingredients: *Bupleurum chinense* (Chaihu), *Scutellaria baicalensis* (Huangqin), *Pinellia ternata* (Banxia), *Codonopsis pilosula* (Dangshen), *Rheum palmatum* (Dahuang), *Zingiber officinale* (Shengjiang), *Ziziphus jujuba* (Dazao), *Poria cocos* (Fuling), *Ostrea gigas* (Muli), and *Platycladus orientalis* (Cebaiye). According to traditional medical theory, these herbs are used to soothe the liver, relieve emotional constraint, strengthen the spleen, and calm the mind. Such effects are considered potentially relevant to the regulation of hair growth within the TCM theoretical framework. From a modern biomedical perspective, some components of HSD have been reported to be involved in pathways related to hair follicle biology. For example, baicalin derived from *Scutellaria baicalensis* can activate the PI3K/AKT pathway, upregulate TGF-β1 and VEGF expression, and promote dermal papilla cell proliferation (Zhang et al., 2022). Extracts from *Platycladus orientalis* leaves have been reported to promote dermal papilla cell proliferation *via* the Akt/GSK3β/β-catenin signaling pathway (Li et al., 2022a). *Zingiber officinale* has been traditionally used for scalp and hair-related disorders, and clinical studies have suggested that oral ginger powder may improve oxidative stress and trace element imbalance in patients with AA (Li et al., 2025). *Poria cocos* has been reported to promote hair follicle cell proliferation and modulate steroid hormone signaling (Zhang et al., 2025). In addition, experimental studies have shown that essential oil from *Ziziphus jujuba* seeds can enhance hair growth parameters in mouse models (Qu, Wu & Guo, 2025), and its extracts have attracted attention for their antioxidant and anti-inflammatory properties in skin and hair care research (Hou, 2020). Although direct clinical evidence for each individual component remains limited, HSD has been applied in clinical practice for AA, and preliminary observations have suggested potential benefits. In this study, we employed bioinformatics approaches to explore the potential molecular mechanisms of HSD, aiming to provide a theoretical and mechanistic basis for future experimental and clinical investigations.

Network pharmacology represents a promising strategy to explore the multi-target, multi-pathway mechanisms of TCM formulations, in alignment with the holistic principles of TCM (Lv et al., 2022; Yu et al., 2024). By integrating bioinformatics, machine learning, and molecular docking techniques, this study aims to systematically investigate the molecular and pathway-level mechanisms through which HSD exerts its therapeutic effects in AA. Bioinformatics, which combines mathematical, statistical, and information science methodologies, plays a crucial role in analyzing biological data to support disease diagnosis and treatment (Ayo et al., 2020). Recent advances in machine learning have demonstrated their potential in identifying key disease-related genes and biomarkers, which can facilitate data mining, disease prediction, and the treatment of AA (Aborode et al., 2025). Weighted gene co-expression network analysis (WGCNA) and immune infiltration analysis can uncover critical genes and immune states associated with clinical features, providing novel insights into the pathophysiology of AA (Wang et al., 2019; Ye et al., 2022). Furthermore, molecular docking and dynamic simulations can model ligand–receptor interactions and dynamic drug-target relationships, shedding light on the structure–activity relationships of potential therapeutic agents (Pinzi & Rastelli, 2019; Wu et al., 2022).

This study employs multiple strategies to explore the molecular mechanisms of Hejie Shengfa Decoction (HSD) in the treatment of alopecia areata (AA). Using network pharmacology, machine learning, immune infiltration analysis, molecular docking, and molecular dynamics simulation, we aim to clarify HSD’s therapeutic action. Our findings will enhance the understanding of HSD’s mechanisms in AA treatment and provide a scientific basis for the clinical application of ethnomedicine and the development of natural treatments for AA.

## Materials & Methods

### Mining of active components and targets of HSD

The active ingredients of the nine medicinal herbs in HSD—*Bupleurum chinense*, *Pinellia ternata*, *Scutellaria baicalensis*, *Rheum palmatum*, *Codonopsis pilosula*, *Zingiber officinale*, *Ziziphus jujuba*, leaves of *Platycladus orientalis*, and the core of *Poria cocos*—were initially retrieved from the Traditional Chinese Medicine Systems Pharmacology Database (TCMSP, version 2.3; https://www.tcmsp-e.com/). Compounds were filtered using the widely accepted criteria of oral bioavailability (OB) ≥ 30% and drug-likeness (DL) ≥ 0.18. Because TCMSP does not comprehensively cover marine-derived ingredients, the active components of oysters were supplemented using the HERB database (HERB 2.0; http://herb.ac.cn/), and duplicate entries were removed. All non-redundant compounds were subsequently evaluated for drug-like properties using SwissADME (current release; https://www.swissadme.ch/) following Lipinski’s rule of five. Molecules satisfying at least three of the five Lipinski criteria were retained as putative active compounds. The isomeric SMILES of these retained compounds were then submitted to SwissTargetPrediction (2019 updated version; http://www.swisstargetprediction.ch/), with the organism set to Homo sapiens. Predicted targets with a probability (likelihood) greater than zero were considered reliable. Finally, all predicted protein targets associated with the active compounds were standardized to official gene symbols using the UniProt Knowledgebase (UniProtKB; https://www.uniprot.org/), and redundant entries were removed.

### Acquisition of targets related to AA

Datasets related to alopecia areata (AA) were retrieved from the Gene Expression Omnibus (GEO) database (http://www.ncbi.nlm.nih.gov/geo/) using the keywords “AA” and “Normal,” with the organism restricted to Homo sapiens. The GSE68801 dataset was selected for subsequent analyses, comprising 36 normal control samples and 63 AA samples. As all data were obtained from publicly accessible repositories, no additional ethical approval or informed consent was required. Differentially expressed genes (DEGs) were identified using the limma package (version 3.60.6) in R (version 4.4.1; R Core Team, 2024). Genes were considered significantly differentially expressed if the absolute log_2_ fold change (—log_2_FC—) was greater than 0.4 and the false discovery rate (FDR)–adjusted *P* value was less than 0.05. Visualization of DEGs, including volcano plots and expression distribution plots, was performed using the ggplot2 package (version 3.5.1). Weighted gene co-expression network analysis (WGCNA) was conducted using the WGCNA package (version 1.72-1) to identify gene modules associated with AA. Prior to network construction, genes with a standard deviation <0.4 across all samples were removed to reduce noise. A signed network was then constructed. The soft-thresholding power (β) was selected from a range of 1 to 20 based on the scale-free topology criterion, with a signed R^2^ ≥ 0.8; β = 7 was chosen as the final soft threshold. The minimum module size was set to 10 genes, and modules with highly similar eigengenes were merged using a cut height of 0.25. Each module was assigned a unique color label. Module eigengenes were correlated with clinical phenotypes (AA *vs.* control) to identify AA-associated modules. For genes within these modules, Module Membership (MM) was calculated to evaluate the correlation between individual genes and their corresponding module eigengenes, while Gene Significance (GS) was used to quantify the association between gene expression and the AA phenotype. Genes with relatively high MM and GS values were considered potential key contributors to AA.

Immune cell infiltration analysis was performed using CIBERSORTx (https://cibersortx.stanford.edu/) with 1,000 permutations to robustly estimate the relative proportions of 22 immune cell types. CIBERSORTx generated an empirical *P* value for each sample, and only samples with *P* < 0.05 were included in downstream analyses. It should be noted that the metadata of the original GSE68801 dataset did not provide explicit batch information that could be used for batch effect correction. Therefore, no additional batch effect adjustment was performed. All samples were processed using the same preprocessing pipeline, and between-array normalization was applied to minimize potential technical variation. This information has been explicitly stated to ensure transparency of the analytical procedures.

### Screening potential targets of HSD in AA treatment

To identify potential therapeutic targets of HSD for the treatment of AA, an intersection analysis was performed among three gene sets: (1) differentially expressed genes (DEGs) between AA and normal controls, (2) genes contained in AA-associated co-expression modules identified by WGCNA, and (3) predicted drug targets of the active components in HSD obtained from the network pharmacology analysis. These three gene lists were imported into OriginPro 2025 (OriginLab Corporation, Northampton, MA, USA), and the built-in Venn Diagram and Set Operations functions were used to calculate their intersections based on official gene symbols. Genes that were simultaneously present in all three datasets were defined as intersecting target genes and regarded as putative core targets of HSD in the treatment of AA. These overlapping targets were subsequently used for PPI network construction and functional enrichment analyses.

### Construction of PPI network and drug-disease interaction network

The final set of intersecting target genes was imported into the STRING database (version 12.0; https://string-db.org/), with the organism restricted to *Homo sapiens* and the minimum required interaction score set to 0.40 (medium confidence). In STRING, only interaction relationships supported by major evidence sources were retained, and disconnected nodes were removed to obtain a clear and stable protein–protein interaction (PPI) network. The resulting PPI network file was then exported from STRING and imported into Cytoscape software (version 3.10.2) for visualization and topological network analysis, enabling the identification of key interactions between the potential drug targets of HSD and AA, and the construction of the HSD–AA interaction network.

### GO and KEGG enrichment analysis and construction of the Component-Target-Pathway network

To explore the potential biological functions and major signaling pathways through which HSD may exert therapeutic effects on AA, the intersecting target genes identified in the previous steps were uploaded to the DAVID database (Database for Annotation, Visualization and Integrated Discovery, version 6.8; https://davidbioinformatics.nih.gov/) for Gene Ontology (GO) functional annotation and Kyoto Encyclopedia of Genes and Genomes (KEGG) pathway enrichment analysis. The species was restricted to Homo sapiens. Enriched GO terms (biological process, cellular component and molecular function) and KEGG pathways with a *p*-value < 0.05 were considered statistically significant.Visualization of the enrichment results, including GO bubble plots and Sankey diagrams for KEGG pathway enrichment, was performed using the Bioinformatics online platform (https://www.bioinformatics.com.cn, accessed on [insert date]). To further elucidate the interrelationships among active components of HSD, potential targets and key signaling pathways, a component–target–pathway network was constructed and visualized in Cytoscape (version 3.10.2; https://www.cytoscape.org/). Active ingredients, targets and pathways were represented as different types of nodes, and their interactions were represented as edges, allowing an integrated view of the multicomponent–multitarget–multipathway characteristics of HSD in the treatment of AA.

### Identification of hub genes through machine learning

To further identify hub genes among the intersecting targets and to screen for features capable of distinguishing patients with AA from healthy controls, three machine learning algorithms were applied: least absolute shrinkage and selection operator (LASSO) logistic regression, support vector machine–recursive feature elimination (SVM–RFE), and Random Forest (RF). All analyses were conducted in R (version 4.4.1). LASSO logistic regression was implemented using the glmnet package (version 4.1-10). The model was fitted with family = “binomial” and alpha = 1, and model tuning was performed by 10-fold cross-validation (nfolds = 10), using type.measure = “deviance” as the optimization criterion. The tuning grid for the penalty parameter *λ* was configured such that the minimum value of log(*λ*) was set to −4. Genes with non-zero coefficients in the optimal LASSO model were retained as candidate features. SVM–RFE was performed using a combination of the e1071 (version 1.7-16), kernlab (version 0.9-33) and caret packages (version 7.0-1). A random seed of 123 was set to ensure reproducibility. Feature ranking and recursive elimination were carried out using caretFuncs as the feature selection function, and the subset of genes corresponding to the minimum cross-validation error was selected as the optimal SVM–RFE gene set. The RF model was constructed using the randomForest package (version 4.7−1.2). Variable importance was quantified by the MeanDecreaseGini index, and genes with MeanDecreaseGini >1 were considered important features and included in the RF-derived gene set. Finally, the intersection of genes selected by LASSO, SVM–RFE, and RF was defined as the set of hub genes. These hub genes were used for subsequent correlation analyses, differential expression validation, and downstream functional investigations.

### Immune infiltration analysis

Immune cell infiltration in AA was evaluated using CIBERSORTx (https://cibersortx.stanford.edu/) with the LM22 signature matrix, which estimates the relative proportions of 22 immune cell types in bulk transcriptome data. The normalized expression matrix of the GSE68801 dataset was uploaded to CIBERSORTx, and the analysis was performed with 1,000 permutations to obtain robust deconvolution results. For each sample, CIBERSORTx provided an empirical *p*-value; only samples with *p* < 0.05 were retained for subsequent immune infiltration analyses to ensure the reliability of the inferred immune cell fractions. All downstream statistical analyses and visualizations were carried out in R (version 4.4.1). Stacked bar plots showing the distribution of immune cell proportions across samples and between groups were generated using the reshape2 package (version 1.4.4) and the ggpubr package (version 0.6.0). Differences in immune cell infiltration between AA and control groups were assessed using the Wilcoxon rank-sum test. In addition, Spearman correlation analysis was performed to evaluate the relationships between hub gene expression levels and the relative abundances of immune cell subsets. The resulting correlation coefficients and *p*-values were visualized as heatmaps using the pheatmap package (version 1.0.12).

### Molecular docking

The three-dimensional structures of human receptor proteins encoded by the core genes were obtained from the RCSB Protein Data Bank (PDB, https://www.rcsb.org/) and pre-processed using PyMOL (v3.1.0). All crystal water molecules and irrelevant crystallographic or hetero-atomic components were removed, while essential cofactors or metal ions required for maintaining the stability of the active-site conformation were retained. Ligand structures were retrieved from the PubChem database based on their CAS numbers in SDF format. Energy minimization was then performed using the MM2 force field in Chem3D (v23.0.1; ChemOffice/ChemDraw Suite 23.0, Revvity Signals), and the optimized structures were subsequently converted into PDB format for docking analysis. Receptor and ligand structures were prepared for docking using AutoDockTools (ADT, v1.5.7), in which polar hydrogens were added, Gasteiger partial charges were assigned, and the resulting files were converted into PDBQT format. Semi-flexible molecular docking was conducted using AutoDock Vina (v1.2.0), treating the receptor as rigid while allowing ligand torsions full flexibility. The docking grid box was centered on the known or predicted binding pocket, and multiple binding conformations were generated for further evaluation. All docking poses and protein–ligand interaction modes were visualized and analyzed using PyMOL (v3.1.0).

### Molecular dynamics simulation

The quercetin–CSF1R complex, which exhibited the lowest binding energy in the molecular docking analysis, was selected for molecular dynamics (MD) simulations. All simulations were performed using GROMACS (version 2023.2). The initial protein–ligand complex structure was prepared using the Solution Builder module of CHARMM-GUI (https://www.charmm-gui.org/), in which the complex was embedded in a rectangular periodic solvent box with a minimum distance of 10 Å between any solute atom and the box boundary. The system was solvated with TIP3P water molecules, and Na^+^ and Cl^−^ ions were added to neutralize the system and to achieve a physiological salt concentration of 0.15 mol/L. The CHARMM36m force field was applied to the protein and ions, and ligand parameters were generated in a CHARMM-compatible format *via* CHARMM-GUI. Topology and coordinate files for the full system were then exported to GROMACS. Energy minimization was performed using the steepest descent algorithm for up to 5,000 steps, with long-range electrostatic interactions treated using the particle mesh Ewald (PME) method. The minimization was considered converged when the maximum force on any atom (emtol) fell below 500 kJ mol^−^^1^ nm^−^^1^. After energy minimization, the system was equilibrated in two stages. First, an NVT ensemble was applied to gradually relax the system at 303.15 K using the velocity-rescaling thermostat, followed by NPT equilibration at 303.15 K and 1 bar using the Parrinello–Rahman barostat, for a total equilibration time of 125 ps. Position restraints were imposed on heavy atoms of the protein–ligand complex during equilibration to stabilize the solute while allowing the solvent and ions to redistribute. The final production MD run was carried out for 100 ns with a time step of 2 fs under NPT conditions. Bond lengths involving hydrogen atoms were constrained using the LINCS algorithm. To ensure the robustness and reproducibility of the results, independent replicate simulations were performed with different initial velocity seeds, and the trajectories were analyzed separately. Structural and dynamic properties (such as RMSD and RMSF) and protein–ligand interaction profiles were extracted and visualized using in-house analysis scripts and DuIvyTools.

## Results

### Identification of drug targets and disease targets

**Figure 1:**
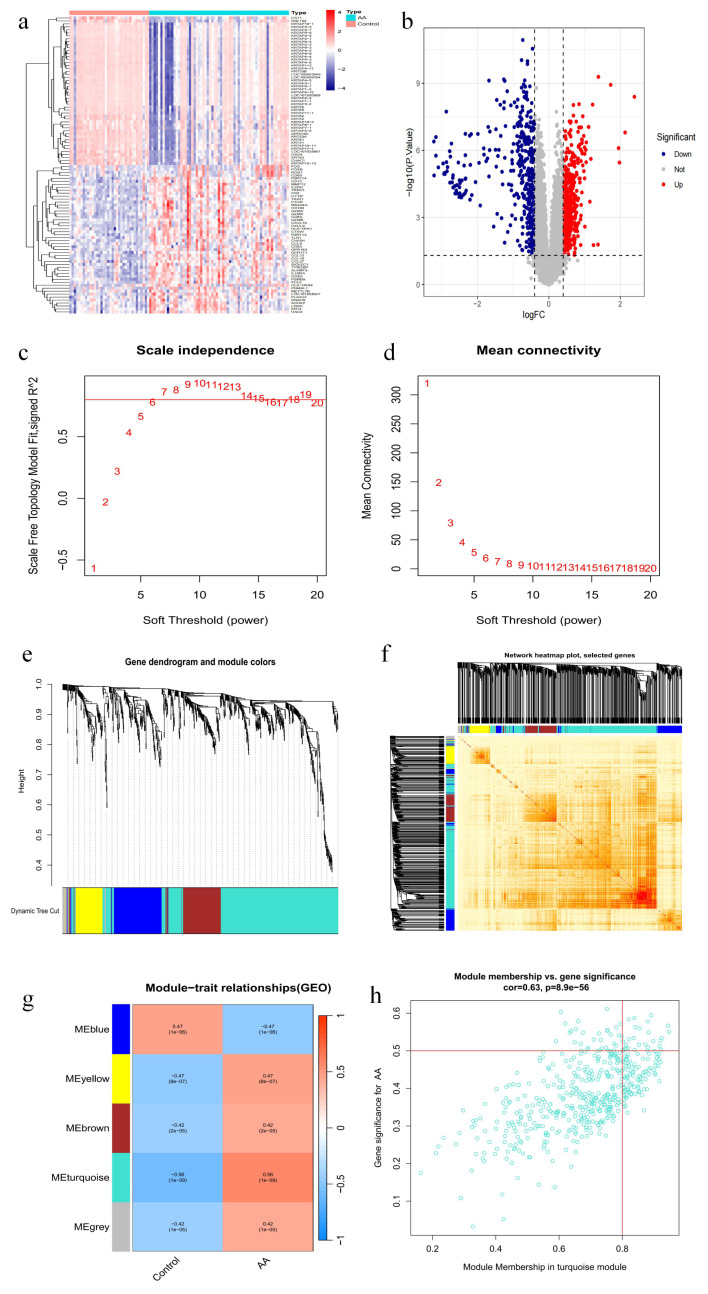
Identification of AA-related genes based on the GSE68801 dataset. Gene expression data were obtained from the Gene Expression Omnibus (GEO) database (GSE68801). (A) Heatmap of differentially expressed genes between AA samples (*n* = 63) and normal controls (*n* = 36). (B) Volcano plot of DEGs identified using limma with —log_2_ fold change (log_2_FC)—> 0.4 and FDR-adjusted *P* < 0.05; log_2_FC is reported as the effect size. (C–D) Determination of the optimal soft-thresholding power for WGCNA based on scale-free topology and mean connectivity. (E) Gene dendrogram and module color assignment obtained by dynamic tree cutting. (F) Gene co-expression network heatmap based on topological overlap. (G) Module–trait relationships between gene modules and the AA phenotype, with correlation coefficients reported as effect sizes and corresponding *P* values shown. (H) Scatter plot showing the correlation between module membership (MM) and gene significance (GS) for AA in the turquoise module.

A total of 96 active ingredients were screened for the active ingredients of the HSD, which were associated with 985 target genes (File S1 and Table S1, respectively). A total of 955 differentially expressed genes were obtained by differential expression analysis of the GSE68801 dataset, including 518 up-regulated genes and 437 down-regulated genes (Figs. 1A and 1B; Table S2). Differential expression analysis was conducted using the GSE68801 dataset obtained from the Gene Expression Omnibus (GEO). This dataset comprised 63 alopecia areata (AA) samples and 36 normal control samples. Differentially expressed genes (DEGs) were identified using the limma package by fitting a linear model with empirical Bayes moderation. After adjusting for multiple testing using the false discovery rate (FDR) method, a total of 955 DEGs were obtained, including 518 upregulated and 437 downregulated genes (Figs. 1A, 1B; Table S2). The magnitude of differential expression was quantified using log_2_ fold change (log_2_FC), reflecting the expression difference between the AA and control groups. To further investigate AA-associated gene co-expression patterns, weighted gene co-expression network analysis (WGCNA) was performed based on the normalized expression matrix of the same GSE68801 dataset. According to the scale-free topology criterion (signed R^2^ ≥ 0.8) together with mean connectivity evaluation, the soft-thresholding power was set to β = 7 (Figs. 1C, 1D). Gene modules were then identified using the dynamic tree cut method, followed by merging of similar modules, resulting in five co-expression modules in total. Among these modules, the turquoise module (MEturquoise) showed the strongest association with the AA phenotype. The hierarchical clustering dendrogram and module color assignment are shown in Fig. 1E, and the corresponding intra-module gene co-expression network is presented in Fig. 1F. Module–trait relationship analysis demonstrated that the turquoise module was most significantly correlated with the AA *versus* control phenotype; the correlation coefficient was used as the effect size, indicating a moderate yet statistically significant association between this module and disease status (Fig. 1G). Further analyses revealed a positive relationship between gene significance (GS) for AA and module membership (MM) within the turquoise module (Fig. 1H), suggesting that genes with higher intramodular connectivity tended to exhibit stronger associations with the AA phenotype. Based on the predefined criteria of relatively high GS and MM values, 492 genes were ultimately selected from the turquoise module as potential AA-associated target genes (Table S3).

### Potential target identification of HSD in AA and development of protein-protein interaction (PPI) and drug-disease networks

**Figure 2:**
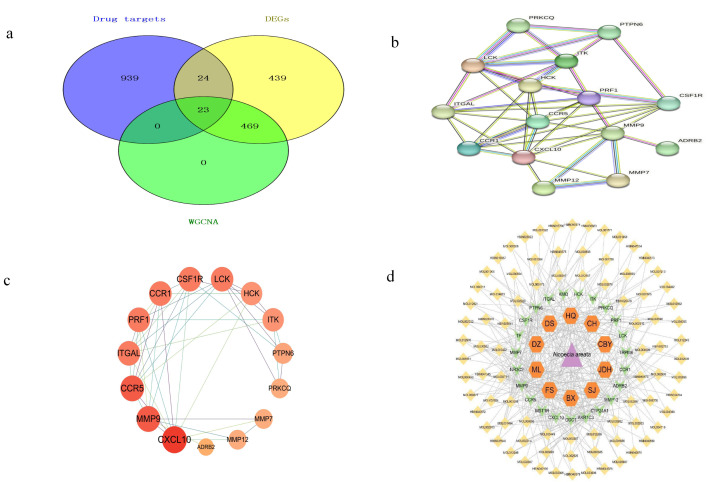
Potential target prediction and construction of PPI and drug-disease network of Reconciliation and HSD in the treatment of AA. (A) Venn diagram of potential target prediction. (B) Original PPI network of intersecting targets. (C) Visualization of PPI networks of intersecting targets. (D) HSD-AA network diagram. Purple triangles indicate AA. Orange hexagonal shapes indicate components in the HSD. Green V-shape indicates intersecting targets. Yellow diamonds indicate active ingredients. The connecting lines indicate the association between the nodes.

The intersection of differentially expressed genes, WGCNA key module genes, and target genes of the active ingredients of HSD was taken to finally obtain 23 intersecting genes (Fig. 2A, Table S4). These genes were considered as potential targets for treating AA with HSD. To further analyze the interactions between the intersecting genes, we selected the top 15 target proteins with the highest display degree values (File S2) to construct a PPI network of the proteins encoded by these genes (Figs. 2B, 2C). In the PPI visualization network, the nodes represent target proteins. The size of the nodes is proportional to the degree value. the higher degree value of the target protein corresponds to the larger nodes, which indicates that it has an important interaction position in the network, and may play a key role in the process of the reconciliation of the HSD for the treatment of AA. To further elucidate the TCM-ingredient-target-disease relationship, the association network of Reconciliation and Hair Regrowth Tang with AA, which contained 10 TCMs, 96 active ingredients, 23 intersecting target genes, and one AA disease node, was constructed (Fig. 2D). The obvious result was that the active ingredients with the highest degree values were soy sterol, quercetin, β-sitosterol, kaempferol, and baicalein, suggesting that these active ingredients inhibit the onset of AA by interacting with the relevant target genes (File S3).

### GO and KEGG pathway enrichment investigation and establishment of the Ingredient-Target-Pathway Network

**Figure 3:**
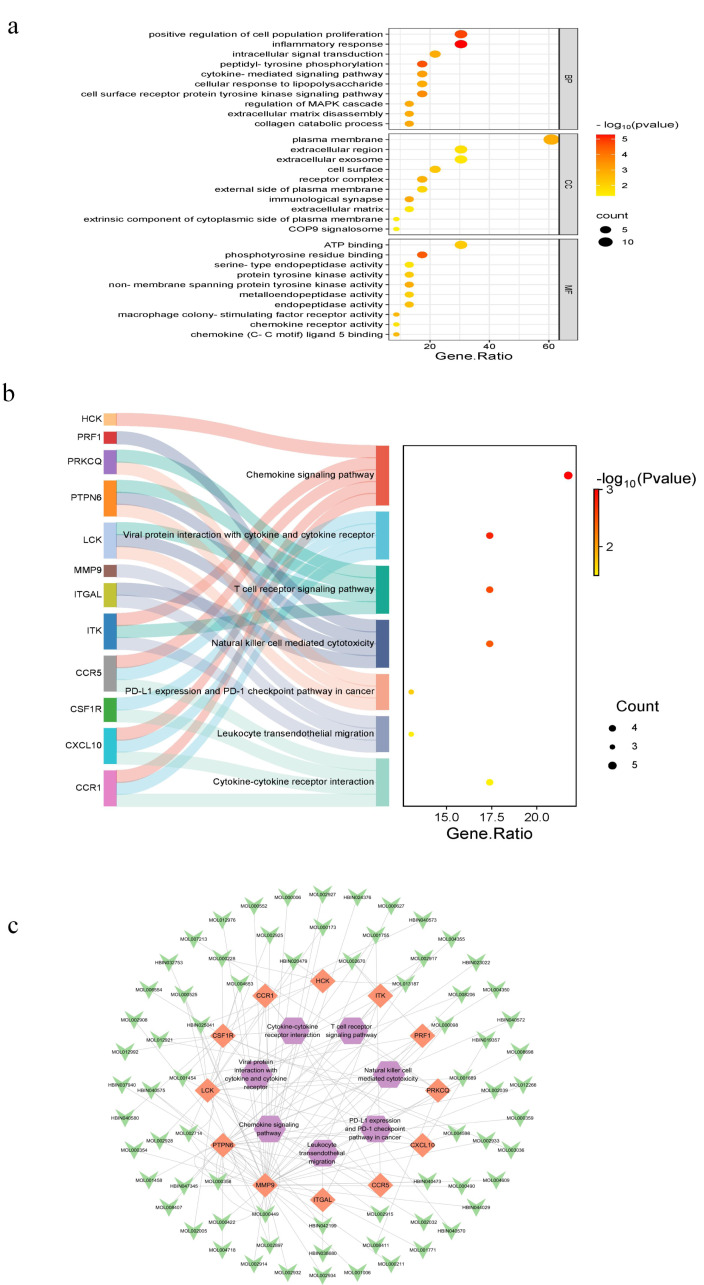
Functional enrichment analysis and component-target-pathway network for the treatment of AA with HSD. (A) Bubble diagram of GO analysis. (B) Sangi bubble diagram of KEGG analysis. (C) Component-target-pathway network diagram of HSD for the treatment of AA. Note: Green V shapes indicate ingredients of HSD. Orange diamonds indicate intersecting targets. Purple hexagons indicate signaling pathways. Connecting lines indicate the association between nodes.

23 intersecting target genes were analyzed for GO and KEGG pathway enrichment. The results of GO analysis showed that the biological processes (BP) in which potential target genes are involved are mainly related to the positive regulation of cell population proliferation, inflammatory response, intracellular signaling, tyrosine phosphorylation, and cytokine-mediated signaling pathways (Fig. 3A). Regions significantly enriched for cellular components (CC) include the plasma membrane, extracellular regions, extracellular exosomes, the cell surface, and receptor complexes. Molecular function (MF) enrichment showed that the main functions involved ATP binding, phosphotyrosine residue binding, serine-type endopeptidase activity, and protein tyrosine kinase activity. KEGG pathway enrichment analysis showed that these intersecting target genes were mainly enriched in chemokine signaling pathways, viral protein-cytokine and their receptor interactions, T cell receptor signaling pathways, natural killer cell-mediated cytotoxicity, and cytokine-cytokine receptor interactions (Fig. 3B). Based on the KEGG analysis of the significantly enriched pathways, a “component-target-pathway network diagram” was constructed to treat of AA with HSD (Fig. 3C).

### Identification of core target genes through machine learning

**Figure 4:**
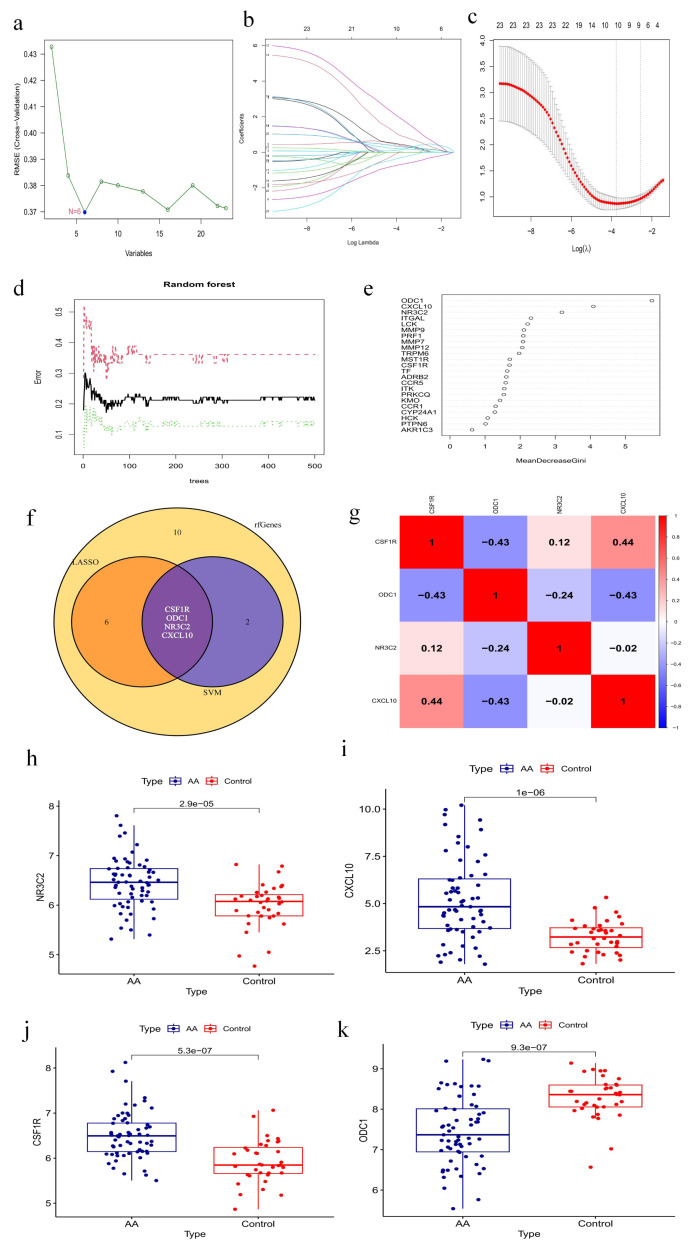
Identification of core AA-related genes using machine learning approaches based on the GSE68801 dataset. (A) Cross-validation curve of LASSO logistic regression based on the GSE68801 dataset (*n* = 99; 63 AA and 36 controls). (B) LASSO coefficient profiles of candidate genes as a function of log(*λ*). (C) Selection of the optimal *λ* by 10-fold cross-validation; error bars represent ± 1 standard error. (D) Random Forest error rate as a function of the number of trees. (E) Variable importance ranking based on MeanDecreaseGini values. (F) Venn diagram showing the overlap of genes selected by LASSO, SVM-RFE, and Random Forest algorithms. (G) Spearman correlation heatmap of the four core genes based on all samples (*n* = 99); numbers indicate correlation coefficients. (H–K) Expression levels of NR3C2, CXCL10, CSF1R, and ODC1 in AA (*n* = 63) and control samples (*n* = 36). Differences between groups were assessed using the Wilcoxon rank-sum test, and exact *p*-values are shown.

Based on the GSE68801 dataset (63 AA samples and 36 normal controls), three machine learning algorithms—Random Forest (RF), Support Vector Machine–Recursive Feature Elimination (SVM-RFE), and Least Absolute Shrinkage and Selection Operator (LASSO)—were applied to identify core target genes associated with alopecia areata. Model performance was evaluated using classification accuracy as the primary performance metric. Using the SVM-RFE algorithm, six candidate genes (ODC1, CXCL10, NR3C2, CSF1R, MMP12, and HCK) were selected as important features (Fig. 4A). LASSO regression retained ten genes, including PTPN6, MMP9, CSF1R, ODC1, MMP7, NR3C2, ADRB2, MST1R, CXCL10, and TRPM6 (Figs. 4B, 4C). The RF algorithm identified 22 candidate genes based on a mean decrease Gini threshold greater than 1 (Figs. 4D, 4E). Intersection analysis of the three algorithms yielded four shared genes—ODC1, CXCL10, NR3C2, and CSF1R—which were defined as core target genes (Fig. 4F). Correlation analysis demonstrated significant interconnectivity among these four genes (Fig. 4G). Furthermore, expression comparisons between AA and control samples showed that NR3C2, CXCL10, and CSF1R were significantly upregulated in the AA group, whereas ODC1 was significantly downregulated (Figs. 4H–4K). Effect sizes are reflected by group-wise expression differences, and statistical significance was assessed using appropriate hypothesis tests.

### Immune infiltration analysis

**Figure 5:**
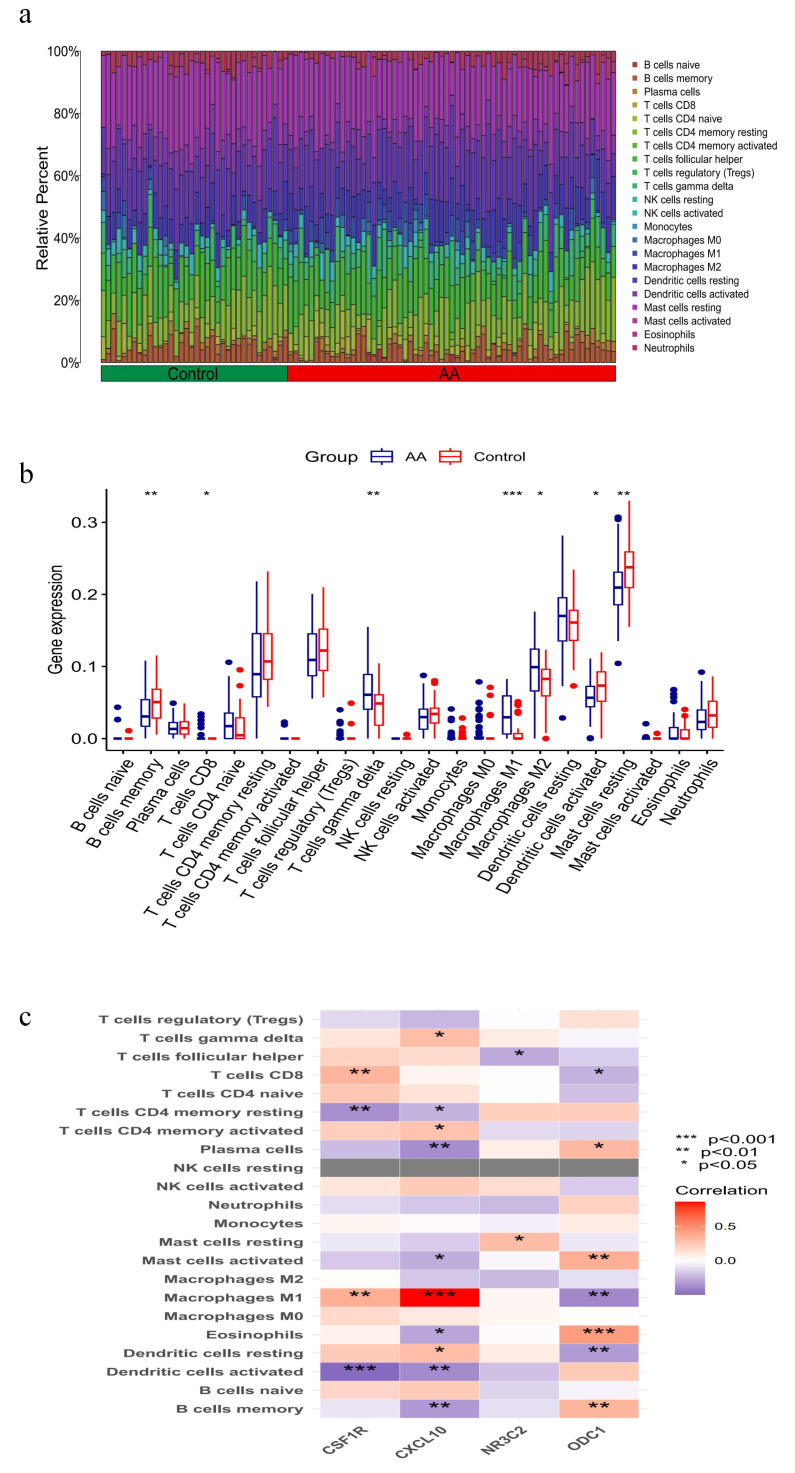
Immune infiltration analysis in alopecia areata. Immune cell infiltration was estimated using the CIBERSORT algorithm based on the GSE68801 dataset, including 63 alopecia areata (AA) samples and 36 control samples. (A) Relative proportions of 22 immune cell types in AA and control samples. (B) Comparison of immune cell infiltration between AA and control groups (AA, *n* = 63; Control, *n* = 36). Effect sizes are expressed as mean differences (AA −Control) with 95% bootstrap confidence intervals (CIs). γ δ T cells (mean difference = 0.043, 95% CI [0.018–0.069]) and M1 macrophages (mean difference = 0.071, 95% CI [0.044–0.098]) showed increased infiltration in AA samples. (C) Correlations between core genes and immune cell infiltration levels, shown as Spearman correlation coefficients. Asterisks indicate statistical significance (*P* < 0.05, * *P* < 0.01, ** *P* < 0.001).

The infiltration of 22 immune cell types in each sample from the GSE68801 dataset was first visualized using the CIBERSORT algorithm (Fig. 5A). Subsequently, immune cell infiltration levels were compared between alopecia areata (AA) samples and non-AA control samples (Fig. 5B). The results showed that the proportions of CD8 T cells, γδ T cells, M1-type macrophages, and resting dendritic cells were significantly higher in AA samples than in controls. Notably, γδ T cells exhibited a mean difference of 0.043 (95% confidence interval (CI) [0.018–0.069], *P* < 0.01), and M1-type macrophages showed a mean difference of 0.071 (95% CI [0.044–0.098], *P* < 0.001), indicating markedly increased infiltration in the AA group. In contrast, the proportions of memory B cells, activated dendritic cells, and resting mast cells were significantly reduced in AA samples. Specifically, memory B cells displayed a negative mean difference of −0.032 (95% CI [−0.051 to −0.014], *P* < 0.01), suggesting decreased infiltration relative to controls. In addition, correlation analysis was performed to explore the relationships between the four core target genes and immune cell infiltration (Fig. 5C). The results demonstrated that CSF1R was significantly negatively correlated with activated dendritic cells, CXCL10 was significantly positively correlated with M1-type macrophages, ODC1 showed a significant positive correlation with eosinophils, and NR3C2 was negatively correlated with follicular helper T cells but positively correlated with resting mast cells.

### Molecular docking

**Figure 6:**
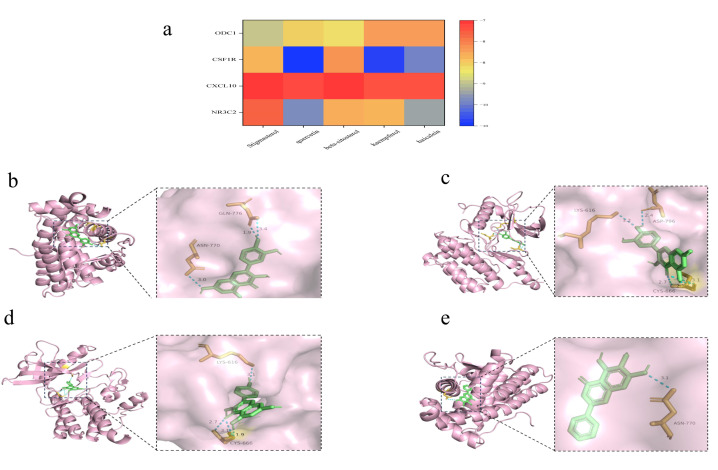
Molecular docking analysis of the components of HSD with target center proteins. (A) Thermogram of the binding energy (kcal/mol) between the active ingredient and the target center protein. (B) Docking plot of quercetin against NR3C2 with a binding energy of −9.5 kcal/mol. (C) Docking plot of quercetin against CSF1R with a binding energy of −10.2 kcal/mol. (D) Docking plot of kaempferol against CSF1R with a binding energy of −10.1 kcal/mol. (E) Docking diagram of baicalein *versus* NR3C2 with a binding energy of −9.3 kcal/mol.

We performed a molecular docking analysis to assess the binding ability of the active ingredients of HSD to the core target genes. The structure files of the four core target genes were obtained from the Protein Data Bank (PDB) as NR3C2 (3WFF), CXCL10 (1O80), CSF1R (6N33), and ODC1 (7U6P) for receptor protein preparation. The five active ingredients with the highest degree values were selected as ligands, including stigmasterol, quercetin, β-sitosterol, kaempferol, and baicalein. Binding energies less than 0 kcal/mol indicated effective binding, and less than or equal to −7.0 kcal/mol indicated tight binding (Fig. 6A). The results showed that all five active ingredients had significant binding potential to the core target genes, with binding energies below −7 kcal/mol. Typical tight binding active target-ingredient interactions are shown in Figs. 6B–6E.

### Dynamic simulation

#### Root mean square deviation

**Figure 7:**
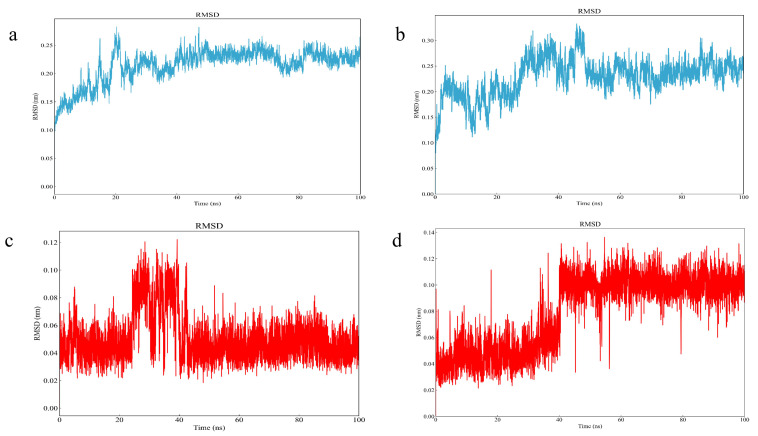
RMSD analysis of the CSF1R–quercetin complex. (A) Time evolution of the RMSD of the CSF1R protein backbone in the first MD simulation. (B) Time evolution of the RMSD of the CSF1R protein backbone in the second MD simulation. (C) Time evolution of the RMSD of quercetin in the first MD simulation. (D) Time evolution of the RMSD of quercetin in the second MD simulation.

RMSD, or Root Mean Square Deviation, is a widely used metric to assess the structural similarity between different conformations of a molecule during a simulation. It is typically calculated by aligning the atomic coordinates of one structure to a reference structure and computing the square root of the average of the squared distances between corresponding atoms. As shown in Figs. 7A, 7B, the atomic coordinates of the protein stabilize after 50 ns in both simulations, with no significant fluctuations observed beyond this point. This suggests that the intramolecular energy of the protein was unstable before 50 ns, and a more stable spatial conformation was achieved at 50 ns. In conjunction with the analysis of the small molecule’s RMSD (Figs. 7C, 7D), it was found that the small molecule also reached structural stability after approximately 43 ns. Although minor fluctuations occurred between 25–43 ns, the variation remained below 0.2 nm (ΔRMSD < 0.2 nm), indicating that the conformational change of the small molecule was minimal. These findings collectively suggest that the binding between the protein and the ligand is stable.

#### Root mean square fluctuation

**Figure 8:**
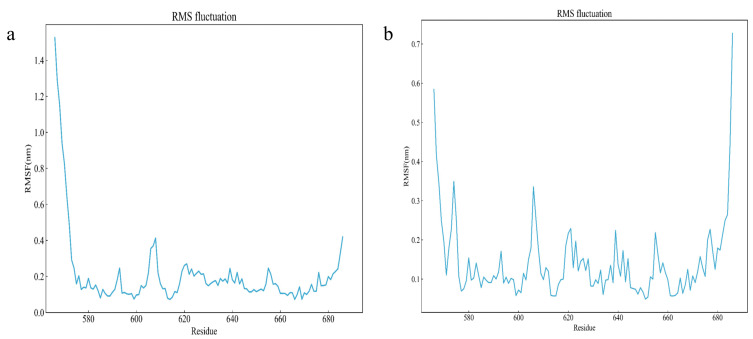
RMSF analysis of CSF1R residues during MD simulations. (A) RMSF profile from the first simulation, showing high flexibility at the N- and C-termini and noticeable peaks near residues 570, 607, 620, 640, and 657. (B) RMSF profile from the second simulation, exhibiting a similar pattern with elevated fluctuations at both termini and the same residue regions, while other areas remain relatively stable.

Root Mean Square Fluctuation (RMSF) is a statistical metric used in molecular dynamics simulations to quantify the positional fluctuations of atoms or particles, reflecting the “vibration” extent of an atom relative to its average position. Higher RMSF values indicate greater flexibility or involvement in dynamic processes. By calculating the RMSF of each amino acid residue, stable and flexible regions of the protein chain can be identified, which is crucial for understanding the protein’s function, conformational changes, and dynamic behavior during ligand binding. The two RMSF analyses (Figs. 8A, 8B) show that the amino acid residues at the N- and C-termini exhibit larger fluctuations, primarily due to the lack of stable covalent bonds and frequent interactions with water molecules. In addition, residues near amino acids 570, 607, 620, 640, and 657 show high RMSF fluctuations, indicating greater flexibility, and suggesting that these regions are unlikely to be binding sites, making it difficult to form stable interactions with small-molecule ligands. In contrast, the RMSF fluctuations in other regions are smaller, implying that these regions may form stable non-covalent interactions with other secondary structures of the same chain or ligand molecules, maintaining a relatively stable conformation. We propose that these regions may represent structurally conserved binding sites.

#### Radius of gyration

**Figure 9:**
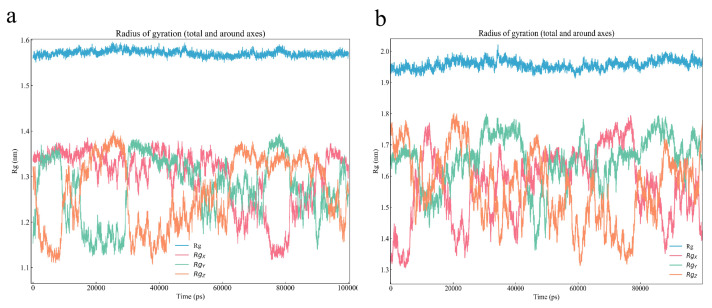
Radius of gyration (Rg) analysis of the protein during molecular dynamics simulations. (A) Rg profile from the first simulation, showing a stable overall Rg with transient and pronounced fluctuations along the x, y, and z axes. (B) Rg profile from the second simulation, exhibiting a similar pattern, where the total Rg remains largely unchanged while dynamic axis-specific fluctuations reflect local structural flexibility.

The Radius of Gyration (RG) is a measure of the extent of mass distribution relative to the geometric center of a molecule, providing information about its size and shape while considering how mass is distributed within space. Unlike simple size measurements, RG reflects the internal flexibility and dynamic properties of the molecule. For a given molecular mass, a more compact structure results in a smaller RG value, while a more extended or relaxed structure yields a larger RG value. In both simulations, the total RG of the protein showed minimal change (ΔRG < 0.2 nm), but significant and transient fluctuations were observed along the XYZ axes, as shown in Figs. 9A and 9B. This suggests that the protein may undergo anisotropic local conformational adjustments. This indicates the potential relative motion between structural domains: although the overall compactness (total RG) remains constant, different domains may undergo coordinated or antagonistic displacements in specific directions. For instance, local bending of α-helices or expansion of β-sheets could lead to transient expansion along particular axes. These observations suggest that the protein may achieve its biological function through a structurally regulated design involving both rigid cores and flexible regions. The rigid core maintains structural stability, while the flexible regions allow necessary conformational adjustments. Such dynamic characteristics may play a crucial role in molecular recognition or signal transduction processes.

#### Hydrogen bond

**Figure 10:**
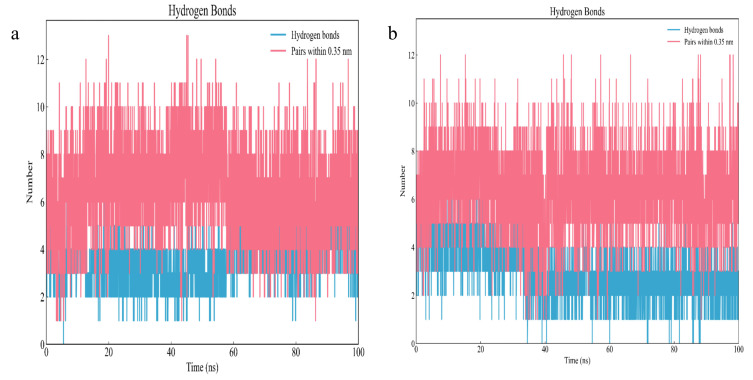
Hydrogen bond and short-range interaction analysis between the ligand and CSF1R during molecular dynamics simulations. (A) Hydrogen bond and short-range contact profiles from the first simulation, showing consistent formation of 2–6 hydrogen bonds and 3–12 close-contact pairs. (B) Hydrogen bond and short-range contact profiles from the second simulation, exhibiting a similar interaction pattern and confirming the reproducibility of stable ligand–protein binding.

Hydrogen bonds are crucial for maintaining the relative positions between molecules and are among the most common and easily formed non-covalent interactions. In biomolecular systems, hydrogen bonds play a key role in processes such as solvation free energy, hydrophobic area, enzyme-substrate complexes, receptor–ligand interactions, and drug design. They help determine the binding modes, affinities, and folding states of macromolecules. We analyzed the hydrogen bond formation between the small molecule and the protein throughout the simulation, and found that in both replicate simulations, the small molecule formed a stable two to six hydrogen bonds with the protein (Figs. 10A, 10B). Furthermore, other short-range interactions (non-hydrogen bonds) also showed significant stability, forming three to 12 interactions, which greatly contribute to the stable binding between the ligand and receptor, maintaining the stability of the system’s conformation.

#### The minimum distance variation trend between pocket amino acids and the small molecule

**Figure 11:**
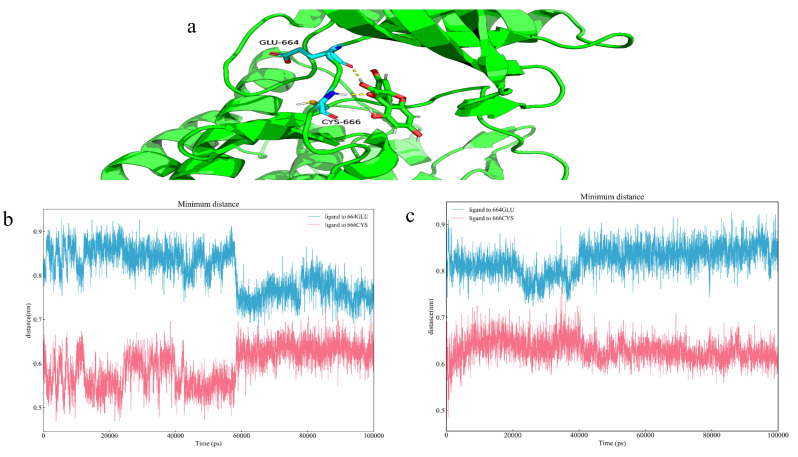
Minimum distance analysis between the ligand and pocket residues GLU664 and CYS666 during MD simulations. (A) Structural view of the binding pocket showing the positions of GLU664 and CYS666 relative to the ligand. (B) Minimum distance profiles from the first simulation, showing small fluctuations and indicating stable ligand binding. (C) Minimum distance profiles from the second simulation, exhibiting a similar trend and confirming reproducible ligand–residue stability.

To further evaluate the stability of the small molecule within the binding pocket, the pocket residues GLU664 and CYS666 were selected for analysis. Specifically, we monitored the minimum distance between the centroids of these residues and the ligand throughout the simulation (Fig. 11A). GLU664 and CYS666 were chosen because they formed hydrogen bonds with the ligand at the end of the simulation, suggesting that such interactions may have been maintained over the course of the trajectory. As shown in Figs. 11B and 11C, the centroid distance exhibited only minor fluctuations across both simulation replicates, indicating that the small molecule remained securely positioned within the binding pocket and did not dissociate. Together with the hydrogen bond analysis, these results provide strong evidence supporting a stable and persistent interaction between the ligand and the protein, effectively anchoring the small molecule within the binding site.

Although global metrics such as RMSD, SASA, hydrogen bond count, and centroid distance may, in certain cases, overemphasize the rigidity of the protein—particularly when compared to the potential flexibility of the ligand—these parameters still serve an important auxiliary role in our study. They are not employed as standalone indicators of binding stability but rather as part of an integrated analytical framework, used in conjunction with more localized and detailed analyses. Specifically, RMSD is utilized to assess the overall structural stability of the protein–ligand complex, while SASA and hydrogen bond counts provide quantitative insights into surface exposure and non-covalent interactions. Centroid distance analysis further aids in evaluating positional changes between the ligand and key residues within the binding pocket. Collectively, these global descriptors offer valuable complementary evidence supporting the stability of the protein–ligand interaction.

## Discussion

This study systematically investigated the biological relevance and potential molecular mechanisms of Hejie Shengfa Decoction (HSD) in alopecia areata (AA). By integrating network pharmacology and machine learning approaches, we identified four core target genes—ODC1, CXCL10, NR3C2, and CSF1R. Molecular docking and molecular dynamics (MD) simulations were then performed to further characterize the interactions between these targets and key bioactive compounds of HSD, including quercetin, stigmasterol, β-sitosterol, kaempferol, and baicalein. The results suggested relatively stable and high-affinity binding patterns. Collectively, these findings indicate that HSD may be involved in multi-target regulatory processes related to immune modulation, inflammatory responses, and hair follicle metabolism; however, they do not constitute evidence of definitive therapeutic efficacy. Importantly, all predicted interactions are derived from computational analyses and should be regarded as hypothesis-generating, requiring subsequent biological validation.

Overall, our findings are consistent with previous studies on the immunopathogenesis of AA and further expand current understanding of potential regulatory pathways. Emotional and psychological disturbances (*e.g.*, anxiety, depression, obsessive-compulsive symptoms, and sleep disorders) are common in patients with AA and may exacerbate disease progression through neuro–immune interactions, forming a vicious cycle (Shakoei et al., 2022; Muntyanu et al., 2023; Dai et al., 2020; Ghalamkarpour et al., 2024; Segalàs et al., 2021; Sánchez-Díaz et al., 2022). In this study, NR3C2, a downstream component of the hypothalamic–pituitary–adrenal (HPA) axis, was identified as a potential key node that may link stress responses to immune imbalance, providing molecular-level support for the TCM concept that emotional dysregulation may contribute to AA pathogenesis (Marie et al., 2007). In addition, CXCL10, a chemokine critical for T-cell recruitment, has been reported to be markedly elevated in both clinical and experimental AA models and can promote CD8^+^ T-cell trafficking toward hair follicles, contributing to the collapse of follicular immune privilege and subsequent follicular damage (Maouia et al., 2017; Xing et al., 2014). This observation aligns closely with the established immunopathogenic feature of AA, in which CD8^+^ NKG2D^+^ T-cell–mediated immune attack plays a central role. ODC1 is the rate-limiting enzyme in the polyamine biosynthesis pathway and is involved in hair shaft elongation and hair follicle regeneration (Soler et al., 1996; Ramot et al., 2010; Kloepper et al., 2010). Previous studies have shown that dysregulated polyamine metabolism can impair hair follicle cycle control and the proliferative capacity of follicular cells, thereby disrupting hair follicle homeostasis (Ramot et al., 2010). In our study, ODC1 was identified as one of the core targets, and subsequent GO/KEGG enrichment analyses further indicated significant enrichment of processes related to cell proliferation and metabolism, providing indirect functional support for the involvement of ODC1. Although there is currently no direct evidence establishing a definitive association between CSF1R and AA, we propose a testable hypothesis that CSF1R may participate in AA-related immune processes by regulating macrophage proliferation and function. In stress-induced hair loss models, increased infiltration of M1 macrophages and the improvement of hair loss following macrophage depletion suggest that macrophage-related pathways may contribute to the pathogenesis of AA (Xiao et al., 2024; Castellana, Paus & Perez-Moreno, 2014). Our MD simulations revealed stable binding between quercetin and CSF1R, providing computational support for this hypothesis; nevertheless, this association remains speculative and requires rigorous functional validation. Importantly, these results should not be interpreted as evidence that HSD treats AA by acting on CSF1R; rather, they serve as a rationale for future mechanistic investigations.

The known pharmacological activities of the core HSD compounds further support the biological plausibility of our findings. Stigmasterol can attenuate chronic inflammation by inhibiting the NF-κB signaling pathway (Si et al., 2024). Quercetin suppresses Th1-type cytokine release and thereby mitigates immune-mediated follicular injury (Yu et al., 2008). β-Sitosterol may help restore Th1/Th2 immune balance and reduce perifollicular T-cell infiltration (Bouic, 2001). Kaempferol alleviates inflammatory injury by inhibiting activation of the MAPK and NF-κB pathways (Kadioglu et al., 2015; Huang et al., 2018). Baicalein has been reported to modulate the JAK–STAT signaling pathway and improve immune imbalance in AA (Kim et al., 2018). Consistently, KEGG pathway enrichment in our study highlighted significant enrichment of cytokine–cytokine receptor interaction, chemokine signaling, and cytotoxic T-cell activation, all of which are key processes in AA immunopathogenesis.

GO and KEGG analyses further suggested that HSD may influence hair follicle regeneration by regulating cell proliferation, immune responses, and inflammatory pathways. GO enrichment emphasized the importance of cellular proliferation in follicular repair, consistent with previous evidence that extracellular matrix dysregulation contributes to follicular instability and hair loss (Subramanya, Coda & Sinha, 2010; McDonagh, Cawood & Messenger, 1990). KEGG enrichment of the chemokine signaling pathway, T-cell receptor (TCR) signaling pathway, and natural killer (NK) cell–mediated cytotoxicity further suggests that both adaptive and innate immune processes are involved in AA development and progression.

Immune infiltration analysis revealed increased levels of CD8^+^ T cells, γδ T cells, M1 macrophages, and resting dendritic cells, together with decreased levels of memory B cells, activated dendritic cells, and resting mast cells, indicating a markedly imbalanced local immune microenvironment in AA. CD8^+^ T cells are considered the major effector cells in AA and can mediate follicular immune attack *via* NKG2D activation (Olayinka & Richmond, 2021). Although the role of γδ T cells in AA remains incompletely understood, their rapid pro-inflammatory responses may contribute to disease acceleration (Bonneville, O’Brien & Born, 2010). Elevated M1 macrophages and their secretion of pro-inflammatory cytokines (IL-1β, TNF-α, and IL-6) support their involvement in amplifying chronic inflammation (Murray & Wynn, 2011). Moreover, changes in CSF1R expression were correlated with shifts in specific immune cell subsets; the biological implications of this observation (*e.g.*, potential effects on macrophage function or antigen presentation) require further functional investigation (Banchereau & Steinman, 1998; Rastogi et al., 2022; Honda & Keith, 2022).

The main strength of this study lies in the integration of network pharmacology, transcriptomics, machine learning, molecular docking, and molecular dynamics simulations to systematically explore the potential mechanisms of traditional Chinese medicine in alopecia areata (AA). In addition, by linking emotional regulation with immune homeostasis, this study provides a conceptual bridge between traditional Chinese medicine theory and modern immunology. Nevertheless, several limitations should be acknowledged. The conclusions of this study are primarily based on computational predictions and publicly available datasets, and there is currently a lack of independent external cohorts with comparable tissue sources and experimental platforms to validate the proposed four-gene panel; therefore, its stability and generalizability remain to be further confirmed. Moreover, the proposed mechanisms—particularly those involving CSF1R—are still largely hypothetical. Although molecular docking and molecular dynamics simulations indicated relatively stable binding tendencies between ligands and targets, systematic validation through *in vitro*, *in vivo*, and clinical studies is still required. Future studies may validate the hypotheses proposed here through both *in vitro* and *in vivo* experiments. At the *in vitro* level, human dermal papilla cells or keratinocyte models may be treated with representative HSD bioactive compounds, followed by qPCR, Western blotting, and gene knockdown or overexpression assays to evaluate the expression and functional changes of CSF1R, CXCL10, ODC1, and NR3C2. At the *in vivo* level, AA or stress-induced hair loss animal models may be employed to systematically assess key outcomes, including immune cell infiltration, macrophage polarization status, and hair follicle cycle dynamics. This validation framework is consistent with experimental designs commonly used to test computational predictions in previous network pharmacology studies (Lv et al., 2022), and may provide direct biological evidence supporting the hypotheses generated in this work.

## Conclusions

This study employed a systematic computational biology approach to explore the potential molecular mechanisms by which Hejie Shengfa Decoction (HSD) may be involved in the pathogenesis of alopecia areata (AA). By integrating network pharmacology, transcriptomic analysis, and machine learning, four core target genes—ODC1, CXCL10, NR3C2, and CSF1R—were identified as potential nodes linking HSD bioactive compounds to immune regulation, inflammatory responses, and hair follicle–related metabolic pathways. Molecular docking and molecular dynamics simulations further suggested stable interaction tendencies between key HSD constituents and these targets, supporting the proposed hypotheses at a structural level. The results suggest that HSD may participate in multi-target regulatory processes associated with immune responses, inflammatory signaling, and hair follicle metabolism. CXCL10 and ODC1 were linked to immune cell recruitment and hair follicle regeneration, respectively, while NR3C2 may represent a potential molecular link between stress responses and immune imbalance. In addition, the proposed involvement of CSF1R highlights a macrophage-associated pathway in AA that warrants further investigation. Importantly, these conclusions are based primarily on computational analyses and publicly available datasets and should therefore be regarded as hypothesis-generating rather than confirmatory evidence of therapeutic efficacy. Further validation through *in vitro* experiments, *in vivo* models, and well-designed clinical studies is required to clarify the biological relevance of HSD in AA. In summary, this study provides a theoretical framework and testable hypotheses that may facilitate future experimental research into AA pathogenesis and the potential integrative role of traditional Chinese medicine in immune-mediated hair disorders.

##  Supplemental Information

10.7717/peerj.21006/supp-1 Supplemental Information 1 All code used in this study (1) Differential analysis code; (2) WGCNA code; (3) Immune infiltration code.

10.7717/peerj.21006/supp-2 Supplemental Information 2 ROC curve for a machine learning model (1)ROC curve of the LASSO logistic regression model; (2) ROC curve of the Random Forest model; (3) ROC curve of the support vector machine (SVM) model.

10.7717/peerj.21006/supp-3 Supplemental Information 3 Codebook

10.7717/peerj.21006/supp-4 Supplemental Information 4 Second molecular dynamics simulation Input data required for the second molecular dynamics simulation:
index.ndx: A file containing the index set of the simulation system, usually used to define the selection of atoms.
md.gro: A GROMACS coordinate file containing the positions of all atoms in the system at a specific time, which is essential for the simulation.
step3_input.pdb: A PDB file used as the input structure for the second simulation step, containing the 3D coordinates of the molecules involved in the simulation.

10.7717/peerj.21006/supp-5 Supplemental Information 5 First molecular dynamics simulation data Input files for the first molecular dynamics simulation, including the structural and parameter files used in the initial molecular dynamics setup.

10.7717/peerj.21006/supp-6 Supplemental Information 6 Supplemental tables related to the analysis presented in the manuscript

## Additional Information and Declarations

### Data Availability

The following information was supplied regarding data availability: The raw data and code are available in the Supplemental Files.
