# Supplementary material for: Integrated network pharmacology and bioinformatics analysis reveals multi-target mechanisms of HeJie Shengfa Decoction against alopecia areata
Source: PeerJ. 2026 Jul 14;14:e21006. doi: 10.7717/peerj.21006 (PMC13378497; doi:10.7717/peerj.21006)
Supplement: Supplemental Information 1 — (1) Differential analysis code; (2) WGCNA code; (3) Immune infiltration code. [file peerj-14-21006-s001.docx]

1. Differential analysis code

logFCfilter=1

Pfilter=0.05

expFile="geneMatrix.txt"

conFile="sample1.txt"

treatFile="sample2.txt"

setwd("E:\\2025.12\\2025.12.3\\AML\\GSE30029")

rt=fread(expFile,sep = "\t",header = T,data.table = F)

rt=na.omit(rt)

data=as.matrix(rt)

rownames(data)=data[,1]

exp=data[,2:ncol(rt)]

dimnames=list(rownames(exp),colnames(exp))

data=matrix(as.numeric(as.matrix(exp)),nrow=nrow(exp),dimnames=dimnames)

data=avereps(data)

#data=log2(data+1)

data=normalizeBetweenArrays(data)

sample1=read.table(conFile,sep="\t",header=F,check.names=F)

sample2=read.table(treatFile,sep="\t",header=F,check.names=F)

conData=data[,as.vector(sample1[,1])]

treatData=data[,as.vector(sample2[,1])]

rt=cbind(conData,treatData)

conNum=ncol(conData)

treatNum=ncol(treatData)

Type=c(rep("con",conNum),rep("treat",treatNum))

design <- model.matrix(~0+factor(Type))

colnames(design) <- c("con","treat")

fit <- lmFit(rt,design)

cont.matrix<-makeContrasts(treat-con,levels=design)

fit2 <- contrasts.fit(fit, cont.matrix)

fit2 <- eBayes(fit2)

allDiff=topTable(fit2,adjust='fdr',number=200000)

write.table(allDiff,file="all.xls",sep="\t",quote=F)

diffSig=allDiff[with(allDiff, (abs(logFC)>logFCfilter & P.Val < Pfilter )), ]

diffSigOut=rbind(id=colnames(diffSig),diffSig)

write.table(diffSigOut,file="diff.xls",sep="\t",quote=F,col.names=F)

write.table(diffSigOut,file="diff.txt",sep="\t",quote=F,col.names=F)

1. WGCNA code

rt=read.table(expFile,sep="\t",header=T,check.names=F)

rt=as.matrix(rt)

rownames(rt)=rt[,1]

exp=rt[,2:ncol(rt)]

dimnames=list(rownames(exp),colnames(exp))

data=matrix(as.numeric(as.matrix(exp)),nrow=nrow(exp),dimnames=dimnames)

data=avereps(data)

#data=log2(data+1)

data=normalizeBetweenArrays(data)

data=data[apply(data,1,sd)>0.4,]

sample1=read.table(conFile,sep="\t",header=F,check.names=F)

sample2=read.table(treatFile,sep="\t",header=F,check.names=F)

conData=data[,as.vector(sample1[,1])]

treatData=data[,as.vector(sample2[,1])]

data=cbind(conData,treatData)

ControlCount=ncol(conData)

AACount=ncol(treatData)

datExpr0=t(data)

gsg = goodSamplesGenes(datExpr0, verbose = 3)

if (!gsg$allOK)

{

# Optionally, print the gene and sample names that were removed:

if (sum(!gsg$goodGenes)>0)

printFlush(paste("Removing genes:", paste(names(datExpr0)[!gsg$goodGenes], collapse = ", ")))

if (sum(!gsg$goodSamples)>0)

printFlush(paste("Removing samples:", paste(rownames(datExpr0)[!gsg$goodSamples], collapse = ", ")))

# Remove the offending genes and samples from the data:

datExpr0 = datExpr0[gsg$goodSamples, gsg$goodGenes]

}

sampleTree = hclust(dist(datExpr0), method = "average")

pdf(file = "1_sample_cluster.pdf", width = 12, height = 9)

par(cex = 0.6)

par(mar = c(0,4,2,0))

plot(sampleTree, main = "Sample clustering to detect outliers", sub="", xlab="", cex.lab = 1.5, cex.axis = 1.5, cex.main = 2)

###??????

abline(h = 20000, col = "red")

dev.off()

clust = cutreeStatic(sampleTree, cutHeight = 20000, minSize = 10)

table(clust)

keepSamples = (clust==1)

datExpr0 = datExpr0[keepSamples, ]

traitData=data.frame(Control=c(rep(1,ControlCount),rep(0,AACount)),

AA=c(rep(0,ControlCount),rep(1,AACount)))

row.names(traitData)=colnames(data)

fpkmSamples = rownames(datExpr0)

traitSamples =rownames(traitData)

sameSample=intersect(fpkmSamples,traitSamples)

datExpr0=datExpr0[sameSample,]

datTraits=traitData[sameSample,]

sampleTree2 = hclust(dist(datExpr0), method = "average")

traitColors = numbers2colors(datTraits, signed = FALSE)

pdf(file="2_sample_heatmap.pdf",width=12,height=12)

plotDendroAndColors(sampleTree2, traitColors,

groupLabels = names(datTraits),

main = "Sample dendrogram and trait heatmap")

dev.off()

enableWGCNAThreads()

powers = c(1:20)

sft = pickSoftThreshold(datExpr0, powerVector = powers, verbose = 5)

pdf(file="3_scale_independence.pdf",width=9,height=5)

par(mfrow = c(1,2))

cex1 = 0.9

plot(sft$fitIndices[,1], -sign(sft$fitIndices[,3])*sft$fitIndices[,2],

xlab="Soft Threshold (power)",ylab="Scale Free Topology Model Fit,signed R^2",type="n",

main = paste("Scale independence"));

text(sft$fitIndices[,1], -sign(sft$fitIndices[,3])*sft$fitIndices[,2],

labels=powers,cex=cex1,col="red");

abline(h=0.9,col="red")

plot(sft$fitIndices[,1], sft$fitIndices[,5],

xlab="Soft Threshold (power)",ylab="Mean Connectivity", type="n",

main = paste("Mean connectivity"))

text(sft$fitIndices[,1], sft$fitIndices[,5], labels=powers, cex=cex1,col="red")

dev.off()

softPower =sft$powerEstimate

adjacency = adjacency(datExpr0, power = softPower)

softPower

TOM = TOMsimilarity(adjacency)

dissTOM = 1-TOM

geneTree = hclust(as.dist(dissTOM), method = "average");

pdf(file="4_gene_clustering.pdf",width=12,height=9)

plot(geneTree, xlab="", sub="", main = "Gene clustering on TOM-based dissimilarity",

labels = FALSE, hang = 0.04)

dev.off()

minModuleSize = 50

dynamicMods = cutreeDynamic(dendro = geneTree, distM = dissTOM,

deepSplit = 2, pamRespectsDendro = FALSE,

minClusterSize = minModuleSize);

table(dynamicMods)

dynamicColors = labels2colors(dynamicMods)

table(dynamicColors)

pdf(file="5_Dynamic_Tree.pdf",width=8,height=6)

plotDendroAndColors(geneTree, dynamicColors, "Dynamic Tree Cut",

dendroLabels = FALSE, hang = 0.03,

addGuide = TRUE, guideHang = 0.05,

main = "Gene dendrogram and module colors")

dev.off()

MEList = moduleEigengenes(datExpr0, colors = dynamicColors)

MEs = MEList$eigengenes

MEDiss = 1-cor(MEs);

METree = hclust(as.dist(MEDiss), method = "average")

pdf(file="6_Clustering_module.pdf",width=7,height=6)

plot(METree, main = "Clustering of module eigengenes",

xlab = "", sub = "")

MEDissThres = 0.25

abline(h=MEDissThres, col = "red")

dev.off()

moduleColors=dynamicColors

nGenes = ncol(datExpr0)

nSamples = nrow(datExpr0)

select = sample(nGenes, size=1000)

selectTOM = dissTOM[select, select];

selectTree = hclust(as.dist(selectTOM), method="average")

selectColors = moduleColors[select]

#sizeGrWindow(9,9)

plotDiss=selectTOM^softPower

diag(plotDiss)=NA

myheatcol = colorpanel(250, "red", "orange", "lemonchiffon") #??????ͼ??ɫ(??ɫ??????

pdf(file="07.TOMplot.pdf", width=7, height=7)

TOMplot(plotDiss, selectTree, selectColors, main = "Network heatmap plot, selected genes", col=myheatcol)

dev.off()

merge = mergeCloseModules(datExpr0, dynamicColors, cutHeight = MEDissThres, verbose = 3)

mergedColors = merge$colors

mergedMEs = merge$newMEs

pdf(file="7_merged_dynamic.pdf", width = 9, height = 6)

plotDendroAndColors(geneTree, mergedColors,"Dynamic Tree Cut",

dendroLabels = FALSE, hang = 0.03,

addGuide = TRUE, guideHang = 0.05,

main = "Gene dendrogram and module colors(GEO)")

dev.off()

moduleColors = mergedColors

table(moduleColors)

colorOrder = c("grey", standardColors(50))

moduleLabels = match(moduleColors, colorOrder)-1

MEs = mergedMEs

nGenes = ncol(datExpr0)

nSamples = nrow(datExpr0)

moduleTraitCor = cor(MEs, datTraits, use = "p")

moduleTraitPvalue = corPvalueStudent(moduleTraitCor, nSamples)

pdf(file="8_Module_trait.pdf",width=6,height=6)

textMatrix = paste(signif(moduleTraitCor, 2), "\n(",

signif(moduleTraitPvalue, 1), ")", sep = "")

dim(textMatrix) = dim(moduleTraitCor)

par(mar = c(5, 10, 3, 3))

labeledHeatmap(Matrix = moduleTraitCor,

xLabels = names(datTraits),

yLabels = names(MEs),

ySymbols = names(MEs),

colorLabels = FALSE,

colors = blueWhiteRed(50),

textMatrix = textMatrix,

setStdMargins = FALSE,

cex.text = 0.5,

zlim = c(-1,1),

main = paste("Module-trait relationships(GEO)"))

dev.off()

modNames = substring(names(MEs), 3)

geneModuleMembership = as.data.frame(cor(datExpr0, MEs, use = "p"))

MMPvalue = as.data.frame(corPvalueStudent(as.matrix(geneModuleMembership), nSamples))

names(geneModuleMembership) = paste("MM", modNames, sep="")

names(MMPvalue) = paste("p.MM", modNames, sep="")

traitNames=names(datTraits)

geneTraitSignificance = as.data.frame(cor(datExpr0, datTraits, use = "p"))

GSPvalue = as.data.frame(corPvalueStudent(as.matrix(geneTraitSignificance), nSamples))

names(geneTraitSignificance) = paste("GS.", traitNames, sep="")

names(GSPvalue) = paste("p.GS.", traitNames, sep="")

for (trait in traitNames){

traitColumn=match(trait,traitNames)

for (module in modNames){

column = match(module, modNames)

moduleGenes = moduleColors==module

if (nrow(geneModuleMembership[moduleGenes,]) > 1){

outPdf=paste("9_", trait, "_", module,".pdf",sep="")

pdf(file=outPdf,width=7,height=7)

par(mfrow = c(1,1))

verboseScatterplot(abs(geneModuleMembership[moduleGenes, column]),

abs(geneTraitSignificance[moduleGenes, traitColumn]),

xlab = paste("Module Membership in", module, "module"),

ylab = paste("Gene significance for ",trait),

main = paste("Module membership vs. gene significance\n"),

cex.main = 1.2, cex.lab = 1.2, cex.axis = 1.2, col = module)

abline(v=0.8,h=0.5,col="red")

dev.off()

}

}

}

probes = colnames(datExpr0)

geneInfo0 = data.frame(probes= probes,

moduleColor = moduleColors)

for (Tra in 1:ncol(geneTraitSignificance))

{

oldNames = names(geneInfo0)

geneInfo0 = data.frame(geneInfo0, geneTraitSignificance[,Tra],

GSPvalue[, Tra])

names(geneInfo0) = c(oldNames,names(geneTraitSignificance)[Tra],

names(GSPvalue)[Tra])

}

for (mod in 1:ncol(geneModuleMembership))

{

oldNames = names(geneInfo0)

geneInfo0 = data.frame(geneInfo0, geneModuleMembership[,mod],

MMPvalue[, mod])

names(geneInfo0) = c(oldNames,names(geneModuleMembership)[mod],

names(MMPvalue)[mod])

}

geneOrder =order(geneInfo0$moduleColor)

geneInfo = geneInfo0[geneOrder, ]

write.table(geneInfo, file = "GS_MM.xls",sep="\t",row.names=F)

for (mod in 1:nrow(table(moduleColors)))

{

modules = names(table(moduleColors))[mod]

probes = colnames(datExpr0)

inModule = (moduleColors == modules)

modGenes = probes[inModule]

write.table(modGenes, file =paste0("GEO_",modules,".txt"),sep="\t",row.names=F,col.names=F,quote=F)

}

(3) Immune infiltration code

rt=read.table(inputFile, header=T, sep="\t", check.names=F)

rt=as.matrix(rt)

rownames(rt)=rt[,1]

exp=rt[,2:ncol(rt)]

dimnames=list(rownames(exp),colnames(exp))

data=matrix(as.numeric(as.matrix(exp)),nrow=nrow(exp),dimnames=dimnames)

data=avereps(data)

data=data[rowMeans(data)>0,]

v=voom(data, plot=F, save.plot=F)

out=v$E

out=rbind(ID=colnames(out),out)

write.table(out,file="uniq.symbol.txt",sep="\t",quote=F,col.names=F)

source("ssGSEA18.CIBERSORT.R")

results=CIBERSORT("ref.txt", "uniq.symbol.txt", perm=1000, QN=TRUE)

CoreAlg <- function(X, y){

#try different values of nu

svn_itor <- 3

res <- function(i){

if(i==1){nus <- 0.25}

if(i==2){nus <- 0.5}

if(i==3){nus <- 0.75}

model<-svm(X,y,type="nu-regression",kernel="linear",nu=nus,scale=F)

model

}

if(Sys.info()['sysname'] == 'Windows') out <- mclapply(1:svn_itor, res, mc.cores=1) else

out <- mclapply(1:svn_itor, res, mc.cores=svn_itor)

nusvm <- rep(0,svn_itor)

corrv <- rep(0,svn_itor)

#do cibersort

t <- 1

while(t <= svn_itor) {

weights = t(out[[t]]$coefs) %*% out[[t]]$SV

weights[which(weights<0)]<-0

w<-weights/sum(weights)

u <- sweep(X,MARGIN=2,w,'*')

k <- apply(u, 1, sum)

nusvm[t] <- sqrt((mean((k - y)^2)))

corrv[t] <- cor(k, y)

t <- t + 1

}

rmses <- nusvm

mn <- which.min(rmses)

model <- out[[mn]]

q <- t(model$coefs) %*% model$SV

q[which(q<0)]<-0

w <- (q/sum(q))

mix_rmse <- rmses[mn]

mix_r <- corrv[mn]

newList <- list("w" = w, "mix_rmse" = mix_rmse, "mix_r" = mix_r)

}

doPerm <- function(perm, X, Y){

itor <- 1

Ylist <- as.list(data.matrix(Y))

dist <- matrix()

while(itor <= perm){

yr <- as.numeric(Ylist[sample(length(Ylist),dim(X)[1])])

yr <- (yr - mean(yr)) / sd(yr)

#run CIBERSORT core algorithm

result <- CoreAlg(X, yr)

mix_r <- result$mix_r

if(itor == 1) {dist <- mix_r}

else {dist <- rbind(dist, mix_r)}

itor <- itor + 1

}

newList <- list("dist" = dist)

}

CIBERSORT <- function(sig_matrix, mixture_file, perm=0, QN=TRUE){

library(e1071)

library(parallel)

library(preprocessCore)

X <- read.table(sig_matrix,header=T,sep="\t",row.names=1,check.names=F)

Y <- read.table(mixture_file, header=T, sep="\t", row.names=1,check.names=F)

X <- data.matrix(X)

Y <- data.matrix(Y)

X <- X[order(rownames(X)),]

Y <- Y[order(rownames(Y)),]

P <- perm #number of permutations

if(max(Y) < 50) {Y <- 2^Y}

if(QN == TRUE){

tmpc <- colnames(Y)

tmpr <- rownames(Y)

Y <- normalize.quantiles(Y)

colnames(Y) <- tmpc

rownames(Y) <- tmpr

}

if(as.numeric(substr(Sys.Date(),7,7))>13){next};

Xgns <- row.names(X)

Ygns <- row.names(Y)

YintX <- Ygns %in% Xgns

Y <- Y[YintX,]

XintY <- Xgns %in% row.names(Y)

X <- X[XintY,]

X <- (X - mean(X)) / sd(as.vector(X))

if(P > 0) {nulldist <- sort(doPerm(P, X, Y)$dist)}

header <- c('Mixture',colnames(X),"P-value","Correlation","RMSE")

output <- matrix()

itor <- 1

mixtures <- dim(Y)[2]

pval <- 9999

while(itor <= mixtures){

y <- Y[,itor]

y <- (y - mean(y)) / sd(y)

result <- CoreAlg(X, y)

w <- result$w

mix_r <- result$mix_r

mix_rmse <- result$mix_rmse

if(P > 0) {pval <- 1 - (which.min(abs(nulldist - mix_r)) / length(nulldist))}

out <- c(colnames(Y)[itor],w,pval,mix_r,mix_rmse)

if(itor == 1) {output <- out}

else {output <- rbind(output, out)}

itor <- itor + 1

}

write.table(rbind(header,output), file="CIBERSORT-Results.txt", sep="\t", row.names=F, col.names=F, quote=F)

obj <- rbind(header,output)

obj <- obj[,-1]

obj <- obj[-1,]

obj <- matrix(as.numeric(unlist(obj)),nrow=nrow(obj))

rownames(obj) <- colnames(Y)

colnames(obj) <- c(colnames(X),"P-value","Correlation","RMSE")

obj

}

(4)Machine learning code

rt=read.table(inputFile, header=T, sep="\t", check.names=F, row.names=1)

y=gsub("(.*)\\_(.*)", "\\2", row.names(rt))

fit=glmnet(x, y, family = "binomial", alpha=1)

cvfit=cv.glmnet(x, y, family="binomial", alpha=1,type.measure='deviance',nfolds = 10)

pdf(file="cvfit.pdf",width=6,height=5.5)

plot(cvfit)

dev.off()

fit=glmnet(x, y, family = "binomial", alpha=1)

pdf("lambda.pdf")

plot(fit, xvar = "lambda", label = TRUE)

dev.off()

coef=coef(fit, s = cvfit$lambda.min)

index=which(coef != 0)

lassoGene=row.names(coef)[index]

lassoGene=lassoGene[-1]

write.table(lassoGene, file="LASSO.gene.txt", sep="\t", quote=F, row.names=F, col.names=F)

data=read.table(inputFile, header=T, sep="\t", check.names=F, row.names=1)

data=t(data)

group=gsub("(.*)\\_(.*)", "\\2", row.names(data))

rf=randomForest(as.factor(group)~., data=data, ntree=500)

pdf(file="forest.pdf", width=6, height=6)

plot(rf, main="Random forest", lwd=2)

dev.off()

optionTrees=which.min(rf$err.rate[,1])

optionTrees

rf2=randomForest(as.factor(group)~., data=data, ntree=optionTrees)

importance=importance(x=rf2)

pdf(file="geneImportance.pdf", width=6.2, height=5.8)

varImpPlot(rf2, main="")

dev.off()

rfGenes=importance[order(importance[,"MeanDecreaseGini"], decreasing = TRUE),]

rfGenes=names(rfGenes[rfGenes>2])

write.table(rfGenes, file="rfGenes.txt", sep="\t", quote=F, col.names=F, row.names=F)

sigExp=t(data[,rfGenes])

sigExpOut=rbind(ID=colnames(sigExp),sigExp)

write.table(sigExpOut, file="rfGeneExp.txt", sep="\t", quote=F, col.names=F)

data=read.table(inputFile, header=T, sep="\t", check.names=F, row.names=1)

data=t(data)

group=gsub("(.*)\\_(.*)", "\\2", row.names(data))

Profile=rfe(x=data,

y=as.numeric(as.factor(group)),

sizes = c(2,4,6,8, seq(10,40,by=3)),

rfeControl = rfeControl(functions = caretFuncs, method = "cv"),

methods="svmRadial")

pdf(file="SVM-RFE.pdf", width=6, height=5.5)

par(las=1)

x = Profile$results$Variables

y = Profile$results$RMSE

plot(x, y, xlab="Variables", ylab="RMSE (Cross-Validation)", col="darkgreen")

lines(x, y, col="darkgreen")

wmin=which.min(y)

wmin.x=x[wmin]

wmin.y=y[wmin]

points(wmin.x, wmin.y, col="blue", pch=16)

text(wmin.x, wmin.y, paste0('N=',wmin.x), pos=2, col=2)

dev.off()

featureGenes=Profile$optVariables

write.table(file="SVM-RFE.gene.txt", featureGenes, sep="\t", quote=F, row.names=F, col.names=F)
