# Supplementary material for: Integrated network pharmacology and bioinformatics analysis reveals multi-target mechanisms of HeJie Shengfa Decoction against alopecia areata
Source: PeerJ. 2026 Jul 14;14:e21006. doi: 10.7717/peerj.21006 (PMC13378497; doi:10.7717/peerj.21006)
Supplement: Supplemental Information 3 [file peerj-14-21006-s003.docx]

Translations and standardized English wording for Receiver_Operating_Characteristic_(ROC)_curve.zip

This file provides the translations and standardized English wording for the text appearing in the ROC curve figures included in Receiver_Operating_Characteristic_(ROC)_curve.zip.

No raw data or code is included in this file.

After checking the ROC curve figures, no non-English text was identified in the figures. The text appearing in the figures consists of English labels, model names, abbreviations, AUC values, axis labels, and legend labels. The items below provide the visible figure text and the corresponding standardized English wording.

File 1

File name: 4412cc1a-a11d-405a-9eeb-280387e76c48.png

Figure/model: LASSO

Text appearing in the figure and English translation/standardized wording:

1. ROCCurve（AUC=0.939）

English translation/standardized wording: ROC Curve (AUC = 0.939)

2. Sensitivity

English translation/standardized wording: Sensitivity

3. AUC:0.939

English translation/standardized wording: AUC: 0.939

4. Model (AUC = 0.939 )

English translation/standardized wording: Model (AUC = 0.939)

5. Random (AUC=0.5)

English translation/standardized wording: Random classifier (AUC = 0.5)

6. Bestthreshold

English translation/standardized wording: Best threshold

7. Specificity

English translation/standardized wording: Specificity

8. ROC_curve

English translation/standardized wording: ROC curve

File 2

File name: 3ec58890-ae32-40ce-9485-2532417e5de8.png

Figure/model: Random Forest

Text appearing in the figure and English translation/standardized wording:

1. ROC Curve(AUC=0.857)

English translation/standardized wording: ROC Curve (AUC = 0.857)

2. Sensitiv

English translation/standardized wording: Sensitivity

3. AUC:0.857

English translation/standardized wording: AUC: 0.857

4. RandomForest（AUC=0.857)

English translation/standardized wording: Random Forest (AUC = 0.857)

5. Randomclassifier

English translation/standardized wording: Random classifier

6. Best threshold（0.66）

English translation/standardized wording: Best threshold (0.66)

7. 1-Specificity

English translation/standardized wording: 1 - Specificity

8. ROC curve

English translation/standardized wording: ROC curve

File 3

File name: 4c2a08c3-f7ce-4088-ae2a-9514a2c92ecd.png

Figure/model: Support Vector Machine, SVM

Text appearing in the figure and English translation/standardized wording:

1. SVMROCCurve(AUC=0.992）

English translation/standardized wording: SVM ROC Curve (AUC = 0.992)

2. Sensitivity

English translation/standardized wording: Sensitivity

3. AUC:0.992

English translation/standardized wording: AUC: 0.992

4. SVM(AUC=0.992)

English translation/standardized wording: Support Vector Machine (SVM) (AUC = 0.992)

5. Randomclassifier

English translation/standardized wording: Random classifier

6. 1-Specificity

English translation/standardized wording: 1 - Specificity

7. ROC curve

English translation/standardized wording: ROC curve

8. 0.0, 0.2, 0.4, 0.6, 0.8, 1.0

English translation/standardized wording: Axis tick labels/numerical scale values

Notes:

1. No non-English text was identified in the ROC curve figures.

2. The listed items are the visible figure labels and their standardized English wording.

3. The abbreviations are defined as follows:

ROC: Receiver Operating Characteristic

AUC: Area Under the Curve

SVM: Support Vector Machine

LASSO: Least Absolute Shrinkage and Selection Operator

4. This file does not include raw data or code.
