# Supplementary material for: Integrated network pharmacology and bioinformatics analysis reveals multi-target mechanisms of HeJie Shengfa Decoction against alopecia areata
Source: PeerJ. 2026 Jul 14;14:e21006. doi: 10.7717/peerj.21006 (PMC13378497; doi:10.7717/peerj.21006)
Supplement: Supplemental Information 6 [file peerj-14-21006-s006.zip › Supplemental materials information_AA/File S1.docx]

Supplementary Table 1: Information on the 96 Active Components of Hejie Shengfa Decoction

| Abbreviated herbal names and serial numbers | Mol ID/Herb ID | Molecule Name | MW  (g/mol) | OB  (%) | DL |
| --- | --- | --- | --- | --- | --- |
| CH2 | MOL000354 | isorhamnetin | 316.28 | 49.6 | 0.3 |
| CH4 | MOL004598 | 3,5,6,7-tetramethoxy-2-(3,4,5-trimethoxyphenyl)chromone | 432.46 | 31.97 | 0.59 |
| CH5 | MOL004609 | Areapillin | 360.34 | 48.96 | 0.41 |
| CH6 | MOL013187 | Cubebin | 356.4 | 57.12 | 0.63 |
| CH7 | MOL004653 | (+)-Anomalin | 426.5 | 46.05 | 0.65 |
| CH8 | MOL004718 | α-spinasterol | 412.77 | 42.97 | 0.75 |
| CH9 | MOL000490 | petunidin | 317.29 | 30.04 | 0.3 |
| BX1 | MOL001755 | 24-Ethylcholest-4-en-3-one | 412.77 | 36.08 | 0.75 |
| BX2 | MOL002670 | Cavidine | 353.45 | 35.64 | 0.8 |
| HQ1 | MOL001689 | acacetin | 284.28 | 34.97 | 0.24 |
| HQ2 | MOL000173 | wogonin | 284.28 | 30.68 | 0.23 |
| HQ3 | MOL000228 | (2R)-7-hydroxy-5-methoxy-2-phenylchroman-4-one | 270.3 | 55.23 | 0.2 |
| HQ5 | MOL002908 | 5,8,2'-Trihydroxy-7-methoxyflavone | 300.28 | 37.01 | 0.27 |
| HQ6 | MOL002909 | 5,7,2,5-tetrahydroxy-8,6-dimethoxyflavone | 376.34 | 33.82 | 0.45 |
| HQ7 | MOL002910 | Carthamidin | 288.27 | 41.15 | 0.24 |
| HQ8 | MOL002914 | Eriodyctiol (flavanone) | 288.27 | 41.35 | 0.24 |
| HQ9 | MOL002915 | Salvigenin | 328.34 | 49.07 | 0.33 |
| HQ10 | MOL002917 | 5,2',6'-Trihydroxy-7,8-dimethoxyflavone | 330.31 | 45.05 | 0.33 |
| HQ11 | MOL002925 | 5,7,2',6'-Tetrahydroxyflavone | 286.25 | 37.01 | 0.24 |
| HQ12 | MOL002926 | dihydrooroxylin A | 286.3 | 38.72 | 0.23 |
| HQ13 | MOL002927 | Skullcapflavone II | 374.37 | 69.51 | 0.44 |
| HQ14 | MOL002928 | oroxylin a | 284.28 | 41.37 | 0.23 |
| HQ15 | MOL002932 | Panicolin | 314.31 | 76.26 | 0.29 |
| HQ16 | MOL002933 | 5,7,4'-Trihydroxy-8-methoxyflavone | 300.28 | 36.56 | 0.27 |
| HQ17 | MOL002934 | NEOBAICALEIN | 374.37 | 104.34 | 0.44 |
| HQ18 | MOL002937 | DIHYDROOROXYLIN | 286.3 | 66.06 | 0.23 |
| HQ20 | MOL000359 | sitosterol | 414.79 | 36.91 | 0.75 |
| HQ21 | MOL000525 | Norwogonin | 270.25 | 39.4 | 0.21 |
| HQ22 | MOL000552 | 5,2'-Dihydroxy-6,7,8-trimethoxyflavone | 344.34 | 31.71 | 0.35 |
| HQ24 | MOL001458 | coptisine | 320.34 | 30.67 | 0.86 |
| HQ26 | MOL002897 | epiberberine | 336.39 | 43.09 | 0.78 |
| HQ27 | MOL008206 | Moslosooflavone | 298.31 | 44.09 | 0.25 |
| HQ28 | MOL012245 | 5,7,4'-trihydroxy-6-methoxyflavanone | 302.3 | 36.63 | 0.27 |
| HQ29 | MOL012246 | 5,7,4'-trihydroxy-8-methoxyflavanone | 302.3 | 74.24 | 0.26 |
| HQ30 | MOL012266 | rivularin | 344.34 | 37.94 | 0.37 |
| JDH1 | HBIN020479 | chrysophanol | - | - | - |
| JDH2 | HBIN025041 | emodin | - | - | - |
| JDH3 | HBIN042199 | rhein | - | - | - |
| DS1 | MOL001006 | poriferasta-7,22E-dien-3beta-ol | 412.77 | 42.98 | 0.76 |
| DS3 | MOL003036 | ZINC03978781 | 412.77 | 43.83 | 0.76 |
| DS5 | MOL003896 | 7-Methoxy-2-methyl isoflavone | 266.31 | 42.56 | 0.2 |
| DS6 | MOL004355 | Spinasterol | 412.77 | 42.98 | 0.76 |
| DS7 | MOL004492 | Chrysanthemaxanthin | 584.96 | 38.72 | 0.58 |
| DS8 | MOL005321 | Frutinone A | 264.24 | 65.9 | 0.34 |
| DS9 | MOL000006 | luteolin | 286.25 | 36.16 | 0.25 |
| DS10 | MOL006554 | Taraxerol | 426.8 | 38.4 | 0.77 |
| DS11 | MOL008400 | glycitein | 284.28 | 50.48 | 0.24 |
| DS12 | MOL008406 | Spinoside A | 716.95 | 39.97 | 0.4 |
| DS13 | MOL008407 | (8S,9S,10R,13R,14S,17R)-17-[(E,2R,5S)-5-ethyl-6-methylhept-3-en-2-yl]-10,13-dimethyl-1,2,4,7,8,9,11,12,14,15,16,17-dodecahydrocyclopenta[a]phenanthren-3-one | 410.75 | 45.4 | 0.76 |
| DS14 | MOL008411 | 11-Hydroxyrankinidine | 356.46 | 40 | 0.66 |
| SJ3 | MOL001771 | poriferast-5-en-3beta-ol | 414.79 | 36.91 | 0.75 |
| SJ4 | MOL008698 | Dihydrocapsaicin | 307.48 | 47.07 | 0.19 |
| DZ1 | MOL012921 | stepharine | 297.38 | 31.54 | 0.33 |
| DZ2 | MOL012976 | coumestrol | 268.23 | 32.48 | 0.33 |
| DZ3 | MOL012992 | Mauritine D | 342.46 | 89.12 | 0.45 |
| DZ4 | MOL001454 | berberine | 336.39 | 36.86 | 0.77 |
| DZ5 | MOL001522 | (S)-Coclaurine | 285.37 | 42.35 | 0.23 |
| DZ6 | MOL000211 | Mairin | 456.78 | 55.37 | 0.77 |
| DZ9 | MOL004350 | Ruvoside | 390.57 | 36.12 | 0.75 |
| DZ10 | MOL000627 | Stepholidine | 327.41 | 33.1 | 0.54 |
| DZ11 | MOL007213 | Nuciferin | 295.41 | 34.43 | 0.4 |
| DZ12 | MOL000787 | Fumarine | 353.4 | 59.26 | 0.82 |
| DZ13 | MOL002773 | beta-carotene | 536.96 | 37.18 | 0.58 |
| CBY1 | MOL002005 | Hinokinin | 354.38 | 56.5 | 0.64 |
| CBY2 | MOL002032 | DNOP | 390.62 | 40.59 | 0.4 |
| CBY3 | MOL002034 | (5aR,8aS,9R)-9-(3,4,5-trimethoxyphenyl)-5a,6,8a,9-tetrahydro-5H-isobenzofurano[5,6-f][1,3]benzodioxol-8-one | 398.44 | 52.7 | 0.83 |
| CBY4 | MOL002039 | Isopimaric acid | 302.5 | 36.2 | 0.28 |
| FS1 | HBIN014684 | adenine | - | - | - |
| FS2 | HBIN019762 | carotene | - | - | - |
| FS3 | HBIN020984 | citric acid | - | - | - |
| FS4 | HBIN023022 | dehydroeburicoic acid | - | - | - |
| FS5 | HBIN024376 | dodecanoic acid | - | - | - |
| FS6 | HBIN028037 | glucose | - | - | - |
| FS7 | HBIN032753 | Lauric | - | - | - |
| FS8 | HBIN037940 | oleanolic acid | - | - | - |
| FS9 | HBIN038680 | palmitic acid | - | - | - |
| FS10 | HBIN040473 | polyporenic acid | - | - | - |
| FS11 | HBIN040570 | Poricoic acid A | - | - | - |
| FS12 | HBIN040572 | poricoic acid AM | - | - | - |
| FS13 | HBIN040573 | Poricoic acid B | - | - | - |
| FS14 | HBIN040574 | poricoic acid BM | - | - | - |
| FS15 | HBIN040575 | poricoic acid C | - | - | - |
| FS16 | HBIN040579 | poricoic acid E | - | - | - |
| FS17 | HBIN040580 | poricoic acid F | - | - | - |
| FS18 | HBIN047345 | tumulosic acid | - | - | - |
| FS19 | HBIN047534 | Undecanoate | - | - | - |
| ML1 | HBIN015796 | aluminium | - | - | - |
| ML2 | HBIN019357 | calcium phosphate | - | - | - |
| ML3 | HBIN044029 | silicon | - | - | - |
| ML4 | HBIN034204 | magnesium | - | - | - |
| HQ25、DS2 | MOL002879 | Diop | 390.62 | 43.59 | 0.39 |
| BX3、HQ4 | MOL002714 | baicalein | 270.25 | 33.51 | 0.2 |
| CH1、BX5、HQ23、DS4、SJ2、DZ7 | MOL000449 | Stigmasterol | 412.77 | 43.82 | 0.75 |
| CH3、CBY6 | MOL000422 | kaempferol | 286.25 | 41.88 | 0.24 |
| BX4、HQ19、SJ1、DZ8、CBY5 | MOL000358 | beta-sitosterol | 414.79 | 36.91 | 0.75 |
| CH10、DZ14、CBY7 | MOL000098 | quercetin | 302.25 | 46.43 | 0.27 |

Note: The abbreviations used in the table represent the following Chinese herbs: CH - Bupleurum, HQ - Scutellaria baicalensis, BX - Pinellia ternata, DS - Codonopsis pilosula, DH - Rheum palmatum, SJ - Zingiber officinale, DZ - Ziziphus jujuba, FS - Poria, ML - Ostrea gigas, CBY - Platycladus orientalis leaf
