# Supplementary material for: Integrated network pharmacology and bioinformatics analysis reveals multi-target mechanisms of HeJie Shengfa Decoction against alopecia areata
Source: PeerJ. 2026 Jul 14;14:e21006. doi: 10.7717/peerj.21006 (PMC13378497; doi:10.7717/peerj.21006)
Supplement: Supplemental Information 6 [file peerj-14-21006-s006.zip › Supplemental materials information_AA/File S2.docx]

Information of the top 15 targets ranked by degree

| Full Target Name | Target  Acronym | Degree | Betweenness  Centrality | Closeness  Centrality |
| --- | --- | --- | --- | --- |
| C-X-C motif chemokine 10 | CXCL10 | 9 | 0.2124 | 0.625 |
| 67 kDa matrix metalloproteinase-9 | MMP9 | 8 | 0.1630 | 1 |
| C-C chemokine receptor type 5 | CCR5 | 8 | 0.0087 | 0.705 |
| C-C chemokine receptor type 1 | CCR1 | 7 | 0 | 0.65 |
| Macrophage colony-stimulating factor 1 receptor | CSF1R | 7 | 0.0549 | 0.611 |
| Perforin-1 | PRF1 | 7 | 0 | 0 |
| Integrin alpha-L | ITGAL | 7 | 0.1228 | 0.714 |
| Tyrosine-protein kinase Lck | LCK | 7 | 0.2461 | 1 |
| Tyrosine-protein kinase HCK | HCK | 6 | 0.1192 | 0.625 |
| Tyrosine-protein kinase ITK/TSK | ITK | 6 | 0.1274 | 1 |
| Tyrosine-protein phosphatase non-receptor type 6 | PTPN6 | 4 | 0 | 0 |
| Matrilysin | MMP7 | 3 | 0 | 0.666 |
| Macrophage metalloelastase | MMP12 | 3 | 0 | 0.75 |
| Protein kinase C theta type | PRKCQ | 3 | 0 | 1 |
| Beta-2 adrenergic receptor | ADRB2 | 1 | 0 | 0.666 |
