# Supplementary material for: Integrated network pharmacology and bioinformatics analysis reveals multi-target mechanisms of HeJie Shengfa Decoction against alopecia areata
Source: PeerJ. 2026 Jul 14;14:e21006. doi: 10.7717/peerj.21006 (PMC13378497; doi:10.7717/peerj.21006)
Supplement: Supplemental Information 6 [file peerj-14-21006-s006.zip › Supplemental materials information_AA/File S3.docx]

Information of the top 20 active components ranked by degree

| Ingredient Code | Ingredient Name | Degree | Betweenness  Centrality | Closeness  Centrality |
| --- | --- | --- | --- | --- |
| MOL000449 | Stigmasterol | 12 | 0.007313997 | 0.666666667 |
| MOL000098 | quercetin | 12 | 0.005366441 | 0.8 |
| MOL000358 | beta-sitosterol | 10 | 0.004186409 | 0.666666667 |
| MOL000422 | kaempferol | 8 | 0.003174428 | 0.8 |
| MOL002714 | baicalein | 8 | 0.014494585 | 0.8 |
| MOL013187 | Cubebin | 6 | 0.021372784 | 0.857142857 |
| MOL002670 | Cavidine | 6 | 0.020514521 | 0.857142857 |
| MOL000173 | wogonin | 6 | 0.001553916 | 0.857142857 |
| MOL002917 | 5,2',6'-Trihydroxy-7,8-dimethoxyflavone | 6 | 0.001553916 | 0.857142857 |
| MOL002928 | oroxylin a | 6 | 0.001553916 | 0.857142857 |
| HBIN020479 | chrysophanol | 6 | 0.006662 | 0.857142857 |
| HBIN025041 | emodin | 6 | 0.006662 | 0.857142857 |
| MOL004609 | Areapillin | 5 | 0.007776866 | 0.833333333 |
| MOL000228 | (2R)-7-hydroxy-5-methoxy-2-phenylchroman-4-one | 5 | 0.008151587 | 0.833333333 |
| MOL002914 | Eriodyctiol (flavanone) | 5 | 0.001133933 | 0.833333333 |
| MOL002915 | Salvigenin | 5 | 0.001259191 | 0.833333333 |
| MOL002927 | Skullcapflavone II | 5 | 0.002177749 | 0.833333333 |
| MOL002932 | Panicolin | 5 | 0.001133933 | 0.833333333 |
| MOL002934 | NEOBAICALEIN | 5 | 0.002177749 | 0.833333333 |
| MOL000552 | 5,2'-Dihydroxy-6,7,8-trimethoxyflavone | 5 | 0.002177749 | 0.833333333 |
